# Independent Mutations in the *LRP2* Gene Mediating Telescope Eyes and Celestial Eyes in Goldfish

**DOI:** 10.3390/ijms262110625

**Published:** 2025-10-31

**Authors:** Rongni Li, Bo Zhang, Yansheng Sun, Jingyi Li

**Affiliations:** 1 Fisheries Science Institute, Beijing Academy of Agriculture and Forestry Sciences, No. 18 Jiaomen Rd., Beijing 100097, China; lirongni@baafs.net.cn (R.L.); vicsun69@163.com (Y.S.); 2 National Engineering Laboratory for Animal Breeding, China Agricultural University, No. 2 Yuanmingyuan West Rd., Beijing 100193, China; bozhang0606@cau.edu.cn; 3 Key Laboratory of Agricultural Animal Genetics, Breeding and Reproduction of Ministry of Education, College of Animal Science and Technology, Huazhong Agricultural University, No. 1 Shizishan Street, Wuhan 430070, China

**Keywords:** goldfish, celestial eye, *LRP2*, parallel evolution

## Abstract

After intensive artificial selection, the development of celestial eyes in goldfish involves the eyeballs protuberating and turning upwards. Thus, the celestial eye goldfish is an excellent model for both evolutionary and human ocular disease studies. Here, two mapping populations of goldfish with segregating eye phenotypes in the offspring were constructed. Through whole-genome sequencing and RNA-seq for eyeball samples, a premature stop codon in Exon 38 of the *LRP2* gene was identified as the top candidate mutation for the celestial eye in goldfish. Fatty acid metabolism and epidermal cells, especially keratocyte-related functions, were inhibited in the eyeballs of celestial eye goldfish, while inflammatory reactions and extracellular matrix secretions were stimulated. These results suggest the dysfunction of the cornea in the celestial eye goldfish, and the same for the retina, which could be the results of the truncated LRP2 protein. In addition, the same gene, *LRP2*, is in charge of similar phenotypes (celestial eye and telescope eye) in goldfish, but these phenotypes have no shared mutations. In conclusion, the candidate mutation for the celestial eye in goldfish was identified by this study for the first time, and parallel evolutions of similar phenotypes at the molecular level under artificial selection were observed. These findings provide insights into the developmental and evolutionary processes of morphological changes in the eyes of goldfish.

## 1. Introduction

**Figure 1:**
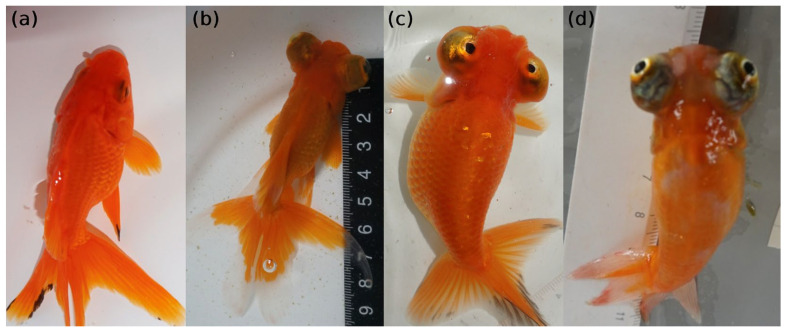
Variety of eye development in goldfish. (**a**) Goldfish with normal eyes (NEs). (**b**) Goldfish with telescope eyes (TEs). (**c**) Goldfish with celestial eyes (CEs). Turning angles of eyeballs are 90°. (**d**) Goldfish with CEs. Turning angles of eyeballs are 45°. The above photos were taken by Rongni Li.

Goldfish are a variety of crucian carp. Beginning in the Song dynasty of ancient China (960–1279 AD), probably from the lower Yangtze River [1], goldfish have been intensively selected for their fascinating morphological traits [2]. Currently, numerous strains of goldfish with various shapes and colors serve as world-famous ornamental fish and also excellent materials for understanding the genetics of morphological traits [3]. Among them, strains with celestial eyes (CEs, also called Sky Gazer, Heavenward Star Gazer, Chotengan, and Deme Ranchu) used to be popular in Chinese palaces because of their enlarged, protuberant, and upward-turning eyes (Figure 1), which were thought of as looking up to the emperor [4]. Now, goldfish with CEs are still considered precious strains. Together with telescope eye goldfish (TEs, which also possess enlarged and protuberant eyes, but the eyeballs are directed toward the side and front, Figure 1), CEs could be utilized as a disease model for eye development [5]. It has been reported that the turning of the eyeballs begins at 3 months of age to 4 months in CE goldfish, with the maximum being 6 months [6]; however, we observed that some of the CE goldfish did not complete the turning of their eyeballs until 10 months of age or were not even not able to turn their eyeballs upwards of 90° by 24 months of age (Figure 1).

There has been a long debate about the genetic relationship between the TE and CE goldfish. Considering their phenotypic similarity, it is reasonable to consider CE goldfish as a variety of TE goldfish with larger upward-turning eyes. However, Komiyama et al. have shown that, according to their mitochondrial genome, TE and CE goldfish evolved independently and originated from goldfish lineages with and without dorsal fins, respectively [7]. A possible earliest reference to the TE goldfish was recorded in 1590, while the CE goldfish appears have originated in the 18th century [8]. Meanwhile, the possibility that gene flow allows the TE mutation to be introduced into goldfish without dorsal fins and contributes to CE has not been excluded. The allelic relationship between TEs and CEs has not been reported yet. What is known is that they are both recessive to normal eyes (NEs) in goldfish [9].

The mutation of TEs was denoted as *d* [9]. In 2020, Kon et al. reported three causal mutations of TEs through GWAS, i.e., a retrotransposon 13 kb insertion in Intron 45 of *lrp2a* (*low-density lipoprotein receptor-related protein 2a*) and two other nonsense mutations for the same gene (Exon 51 and Exon 73) [10]. In addition, gene editing of *lrp2a* via CRISPR/Cas9, which resulted in truncated proteins in two different goldfish strains (287 and 395 AA, respectively, compared with the original LRP2A protein with 4653 AA), both created the TE phenotype [11]. This provides compelling evidence that the incomplete form of LRP2A is responsible for TEs in goldfish. However, it is unknown if TEs and CEs share the same causal mutation(s) at the molecular level.

Through the building of mapping populations and a breed panel, diagnostic tests, whole-genome sequencing (WGS), and RNA-seq analysis, the current study revealed that the CE mutation affects the same gene but does not share the same causal mutations as the TE. These findings provide clues to solve previous debates, while also showing that, under artificial selection for CEs and TEs, parallel evolution occurred at the molecular level.

## 2. Results

### 2.1. Inheritance Pattern of CE in Goldfish

In a mating between CE goldfish, 1277 offspring were obtained, including 1274 CE goldfish with various turning angles of their eyeballs, and only 3 had NEs. The mapping populations were initiated by these three fish. Among them, one NE male was referred to as NEM, and the other two NE females were referred to as NEF1 and NEF2.

**Figure 2:**
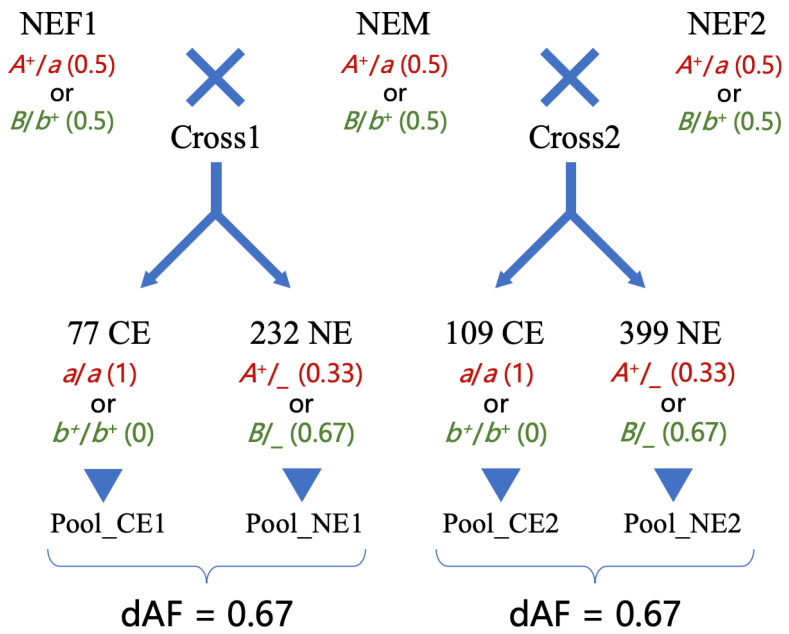
Schematic diagram of the mapping populations. NEF1, NEF2, and NEM are the 3 parental goldfish with NEs, while F and M refer to female and male, respectively. The two matings produced 77 offspring with CEs, 232 with NEs, and 109 with CEs, 399 with NEs, respectively. Four DNA pools were constructed from them. The red letters describe the assumption of a recessive mutation (*a*) causing CEs to be segregated in the offspring, while the green letters are for the different possible scenarios of a dominant inhibiting mutation (*B*) for CEs segregated in the offspring. The numbers in brackets indicate the allelic frequencies of the mutant allele (*a* or *B*). dAF stands for differences in allelic frequencies between two pools.

Mating between NEM and NEF1 (Cross1) was carried out, which produced 309 goldfish (Figure 2). Among them, 77 fish showed different degrees of CEs (59 fish were 90° turned upwards, 12 fish were 80–90°, 4 fish were 45–80°, and 2 fish were 15–45°), and the other 232 fish had NEs. The number of CEs and NEs fish in the offspring of Cross1 matched a 1:3 ratio (*p* > 0.05). In the mating between NEM and NEF2 (Cross2), 634 goldfish were obtained, including 109 with CEs (Figure 2). All these CE fish showed large turning angles of their eyeballs (64 fish were 90° turned upwards, 32 fish were 80–90°, and 13 fish were 45–80°). In addition to CEs, 126 fish with a slightly TE phenotype were also observed in Cross2. However, they failed to develop compete TEs, as their eyes were slightly protuberant without turning upwards. Those unexpected TE fish were excluded from our analysis. The other 399 fish in the offspring of Cross2 had NEs. Thus, the CE and NE fish in Cross2 also matched a 1:3 ratio (*p* > 0.05). These data suggest that a single autosomal recessive gene is responsible for CEs in our mapping populations.

### 2.2. Chromosome 9 Is Associated with CE

For samples from the offspring of Cross1, two DNA pools were constructed based on the shared phenotypes (Pool_CE1, pooling of all 59 CE fish with eyeballs 90° turned upwards, and Pool_NE1, pooling of 80 randomly selected NE fish). Another two DNA pools (Pool_CE2, *n* = 64, and Pool_NE2, *n* = 80) were also constructed using samples from Cross2 with the same criteria of selecting samples for pooling. A total of 1.9 billion reads were obtained by sequencing these four pools. After aligning to the goldfish genome assembly reported by Chen et al. [12], the average mapping rate is 99.34%. The three parental goldfish with NEs were also sequenced individually, which generated 532 million reads with a 99.30% average mapping rate.

Since the CE is recessive and the reference genome we used was from a goldfish with NEs [13], such putative mutation was denoted by an *a* allele in our mapping populations. Thus, the mutant allele (*a*) should be fixed in Pool_CE1 and Pool_CE2 (as red font in Figure 2). Alternatively, the entire mapping populations could be fixed for the CE mutation, but a dominant inhibitor mutation (denoted by the *B* allele in Figure 2) for CEs was segregated. In this case, the wild-type allele (*b*^+^) should be fixed in Pool_CE1 and Pool_CE2 (as blue font in Figure 2). Under these two assumptions, the theoretic allelic frequencies of the mutant allele for each of the parent should be 0.5; for Pool_CE should be 1 or 0, respectively; and for Pool_NE should be 0.33 or 0.67, respectively (since in the offspring, 25% were homozygous mutants, 50% were heterozygotes, and 25% were homozygous wild-type).

**Figure 3:**
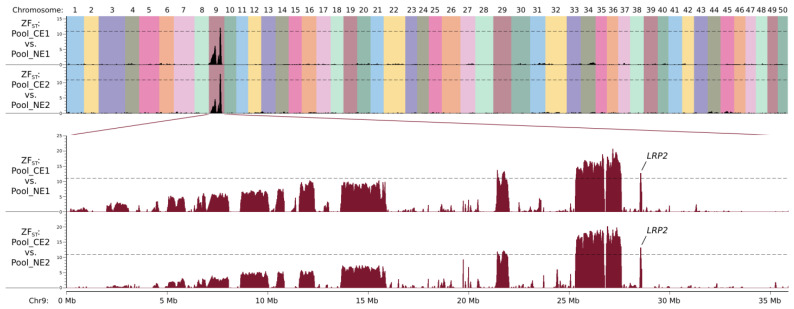
Comparisons of DNA pools revealed selective regions in chromosome 9 associated with celestial eyes (CEs) in goldfish. Pool_CE1 and Pool_NE1 consist of 59 CE and 80 NE (normal eye) fish in the offspring of the first family, respectively. Pool_CE2 and Pool_NE2 consist of 64 CE and 80 NE fish in the offspring of the second family, respectively. Regions with a ZF_ST_ score larger than 11 (dash lines) were considered as candidate regions. Chromosome 9 is roomed-in in the lower panels. The location of the *LRP2* gene is indicated.

By comparing Pool_CE1 and Pool_NE1, regions with ZF_ST_ values larger than 11 were found on chromosome 9 (seven regions with a total size of 2.92 Mb, from 21.39 Mb to 28.63 Mb, see Figure 3 and Table S1; the coordinates refer to the goldfish genome assembly reported by Chen et al. [12], the same below). A total of 8361 SNPs and 2876 other types of variants were defined as candidate mutations by only considering the ones with allelic frequency differences (dAFs) between the two pools larger than 0.5 (as mentioned above, the dAF should be 0.67 in theory (Figure 2); in case of pooling or sequencing error, the threshold of the dAF was set as 0.5); they were heterogeneous in NEM, NEF1, and Pool_NE1 and were homozygous mutant or wild-type in Pool_CE1.

By comparing Pool_CE2 and Pool_NE2, with the same criteria, the candidate region was defined as eight regions on chromosome 9 (with a total size of 3.05 Mb, from 19.73 Mb to 28.63 Mb, see Figure 3 and Table S1; discontinuous candidate regions for both Cross1 and Cross2 could be due to misassembly of the reference genome), and 8368 SNPs and 2874 other mutations were selected for further analysis. The two sets of candidate regions defined by these two comparisons largely overlapped (Figure 3), suggesting that NEF1 and NEF2 share the same causal mutation, and this should be also the same with NEM.

### 2.3. Known TE Causal Mutations Were Not Detected in the CE Goldfish

Since the regions associated with CEs (19.73~28.63 Mb) harbor previously reported TE causal mutations, we first investigated those mutations in our populations. Those mutations include the two nonsense mutations in Exons 51 and 73 of *lpr2aL* [10,11] (corresponding to chr9:28,593,475 and 28,616,634 in the goldfish genome assembly reported by Chen et al. [12]) and the ~13 kb retrotransposon insertion in Intron 45 of the same gene [10]. As a result, the two nonsense mutations were not detected in the mapping populations in this study, while there are five SVs in Intron 45 of *lpr2aL* by viewing the BAM files. Two of them are microsatellite variations, and another two could be transposon elements (Figure S1). Therefore, primers were designed, and Sanger sequencing was applied to further confirm the existence of the ~13 kb insertion in our CE goldfish. In the four CE offspring from our mapping populations, no retrotransposon-specific fragment was amplified. However, the amplification of the entire Intron 45 produced a ~2.5 kb fragment, which is ~0.8 kb longer than the reference sequence but cannot harbor the complete ~13 kb insertion. The sequencing of such PCR products revealed that the leftmost SV in Figure S1 is actually a 33 bp sequence (chr9:28,583,267–28,583,299) replaced by a 116 bp sequence. Other SVs could not be studied with Sanger sequencing because of the microsatellites. However, it is likely that those SVs also added hundreds of base pairs, which contributed to the ~0.8 kb extra sequence. In conclusion, the ~13 kb insertion was not detected in the CE goldfish. Altogether, none of the known TE causal mutations were detected in our CE mapping populations.

### 2.4. CE in Goldfish Is Heterogeneous

Outside of our mapping populations, 50 CE goldfish from five different fish farms were whole-genome sequenced individually, which generated 3.2 billion reads with a 99.38% average mapping rate.

Under the assumption that a single mutation caused all the CE phenotypes, we firstly focused on the mutations that were fixed in all the 52 CE goldfish libraries (Pool_CE1, Pool_CE2, and the 50 CE individuals) from our candidate mutations. As a result, 19 mutations were screened out. However, the majority of them have a low calling rate since quality control for the mutations was not applied. Therefore, after manually confirming their genotypes by viewing the BAM files, most of the allelic frequencies were corrected. As a result, only two SNPs matched the criteria (fixed in the 52 CE goldfish libraries while heterogeneous in the other 5 libraries) and underwent further investigation. These two SNPs (chr9:26,872,019 and 26,872,024) are 5 bps away, located 100 kb and 39 kb downstream of *retsatl* and *Cau.09G0011130*, respectively. They were homozygous WT in all the CE goldfish libraries so that the mutant allele may act as an inhibitor of the CE, if they are functional. They were also possible neutral variants but merely carried by the three NE parents, since they are downstream mutations and far away from the genes.

**Figure 4:**
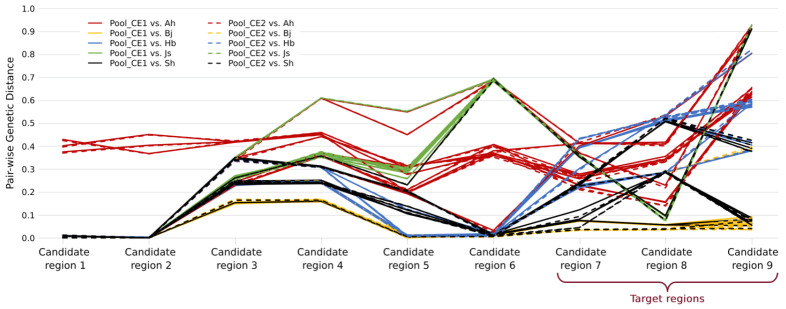
Pair-wise genetic distances between the DNA pools from the mapping populations and individual samples outside of the mapping populations. The DNA pools are Pool_CE1 and Pool_CE2, which are the offspring expressing celestial eyes (CEs) in the mapping populations. The individual CE samples are from 5 different fish farms located in different regions in China, i.e., Anhui (Ah), Beijing (Bj), Hebei (Hb), Jiangsu (Js), and Shanghai (Sh) (*n* = 10 for each farm). Candidate regions were previously defined by ZF_ST_ values. After excluding the samples with genetic distances larger than 0.1 for each region, the target mutations (fixed in all the remaining samples) only distributed in candidate regions 7 to 9 and thus were termed as target regions.

Therefore, we considered the alternative assumption that not all the 50 CE goldfish outside of our mapping populations carried the same CE causal mutation. In order to determine which samples and which regions may harbor the same causal mutation with our mapping populations, pair-wise genetic distances between Pool_CE1 or Pool_CE2 and each of the 50 individual samples were calculated for each candidate region (Figure 4). Within each region, the individual samples with any genetic distance larger than 0.1 were excluded for the target mutation screening (Table S2), and the criteria are similar in that target mutations should be fixed in all the remaining CE libraries. As a result, six out of nine candidate regions (candidate regions 1 to 6 in Table S1) were excluded. Although there are still a large number of target mutations (10,146), they are located within a ~3.2 Mb region (chr9:25,346,752–28,589,750, defined as the target region, including 59 annotated genes). It is also clear that not all CE goldfish shared the same IBD (Identity-by-descent) sequence in the target regions (Figure 4).

### 2.5. Putative Functional Mutations Were Identified as Candidates for CE

To account for potential artifacts during sequencing, all SVs in the expanded region, from 24.35 to 29.59 Mb in chromosome 9 (1 Mb upstream and 1 Mb downstream of the target regions), were investigated for their putative functions. Under the combination of Lumpy software and manually double checking, 97 SVs were detected from the seven libraries of the mapping population (three parental samples and four offspring pools). Eventually, two SVs (a 1.5 kb deletion and a 200 kb complex SV possibly involving deletions and inversions) were sifted out. Next, the genotypes of these two SVs in the 50 individual CE samples were determined by viewing the BAM files, which showed that these two SVs still match the criteria of candidates according to Table S2 (in candidate regions 7 and 8, respectively). This 1.5 kb deletion (chr9:25,574,191–25,575,687) is 3.8 kb downstream of an uncharacterized coding gene (*Cau.09G0010620*) and 36.2 kb downstream of *CALCRLA*; the 200 kb SV (and 27,182,149–27,382,584) harbored three annotated genes (*HDAC4*, *TRAF3IP1*, and *TWIST2*).

Among the target mutations (SNPs and small Indels, *n* = 10,146, as defined above), there are 11 frameshift Indels and 6 non-frameshift but coding Indels and 119 nonsynonymous, 1 stopgain, and 1 stoploss SNP. Those mutations involve 18 other annotated genes. Together with the 4 genes probably being affected by the 2 candidate SVs, these 22 genes are defined as candidate genes.

### 2.6. Epidermis-Related Processes, Fatty Acid Metabolisms, and Immune Responses Were Involved in the Formation of CEs

**Figure 5:**
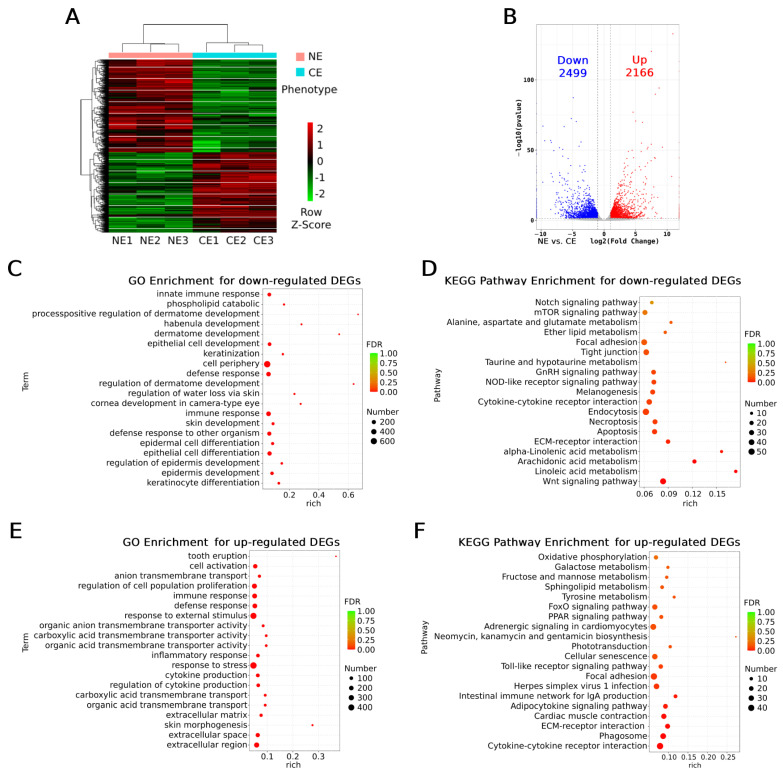
Comparison of expression profiles between eyeballs of NE and CE goldfish via RNA-seq. NE stands for normal eye and CE stands for celestial eye. (**A**) Heatmap for the gene expressions in the 3 NE samples and 3 CE samples. (**B**) Volcano plots showing the differentially expressed genes (DEGs), including up- and down-regulated genes in CE samples. The criteria for DEGs are |log2foldchange| > 1 and adjusted *p*-value < 0.05. Top 20 GO terms (**C**) and top 19 KEGG pathways (**D**) significantly enriched (*p* < 0.05) for the DEGs down-regulated in CE samples. Top 20 GO terms (**E**) and top 20 KEGG pathways (**F**) significantly enriched (*p* < 0.05) for the DEGs up-regulated in CE samples. In each of (**C**–**F**), the *p*-value increases as the terms or pathways are presented from the bottom to the top.

To further narrow down the candidate genes in the target regions, RNA-seq was performed using eyeball samples from NE and CE goldfish (14 months of age). As the heatmap shows, the RNA-seq samples clustered according to their phenotypes, NE or CE (Figure 5A). A total of 4665 differentially expressed genes (DEGs) were detected. Among them, 2499 were down-regulated and 2166 were up-regulated in the CE group (Figure 5B).

The down- and up-regulated DEGs, and all the DEGs (Figure S2), were separately subjected to enrichment analysis by Gene Ontology (GO) and Kyoto Encyclopedia of Genes and Genomes (KEGG) pathways. For the down-regulated DEGs, all of the top five most significant enriched GO terms are epidermal-cell-related processes (keratinocyte differentiation, epidermal cell development, and differentiation, Figure 5C), while three out of the top four most enriched KEGG pathways are fatty acid metabolisms (linoleic acid and arachidonic acid metabolisms, Figure 5D). For the up-regulated DEGs, 6 out of the top 10 most enriched GO terms are also epidermal-cell-related or extracellular terms (also considered as epidermis-related, including extracellular region, space, and matrix and response to external biotic stimulus, Figure 5E); in the rest of the 4 GO terms, 3 are immune-response-related (immune response, defense response, immune system process, ranking the second to fourth most enriched terms, Figure 5E). Immune response pathways were also enriched as the top pathways in the KEGG analysis for the up-regulated DEGs (6 pathways out of the top 10, cytokine–cytokine receptor interaction, phagosome, adipocytokine signaling pathway, intestinal immune network for IgA production, herpes simplex virus 1 infection, and Toll-like receptor signaling pathway, Figure 5F), suggesting that inflammatory reactions may take place in the eyeballs of the CE goldfish. In addition, the PPAR (peroxisome proliferator-activated receptor) signaling pathway was significantly enriched for the up-regulated DEGs (Figure 5F), which is also key for fatty acid metabolism. In addition, terms of cornea development in camera-type eyes and the regulation of water loss via skin were enriched for the down-regulated DEGs together with keratinocyte-related terms (Figure 5C), suggesting dysfunction of corneas in the CE goldfish; the melanogenesis pathway was significantly down-regulated (Figure 5D), while pathways of tyrosine metabolism and phototransduction were up-regulated in the CE goldfish (Figure 5F), suggesting that the retina was also affected. When applying enrichment analysis for all the DEGs, terms including skin morphogenesis, inflammatory response, other extracellular-related terms, pathways involving immune response and fatty acid metabolism, were also enriched (Table S2).

Taken together, our RNA-seq data reveals that epidermis-related functions including extracellular processes were dramatically changed in the eyeballs of the CE goldfish, while fatty acid metabolisms were inhibited and immune responses, especially inflammatory reactions, were stimulated. Functionally important genes in the cornea and retina could be differentially expressed.

### 2.7. LRP2 and Its Coding Mutations Are the Top Candidates

**Figure 6:**
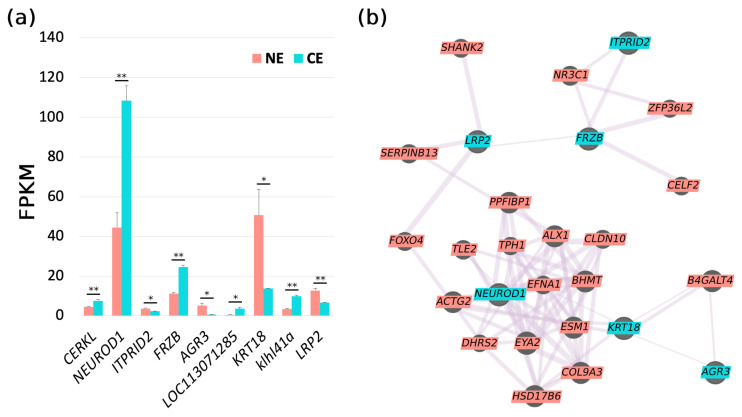
Differentially expressed genes (DEGs) located in the candidate regions of CEs in goldfish. (**a**) Within the candidate regions, 9 DEGs were detected via RNA-seq. NE stands for normal eye and CE stands for celestial eye. Data are represented as mean ± SEM. An asterisk indicates a significant difference (*p* < 0.05), while double asterisks indicate extremely significant differences (*p* < 0.01). Total RNA was extracted from eyeballs, *n* = 3 for each column. (**b**) Predicted gene interactions for the DEGs (blue color, 3 DEGs are not listed since no data or no interaction) and other related genes (pink color) according to the GeneMANIA database.

Among the 59 annotated genes in the target regions, 9 were differentially expressed according to our RNA-seq data. They are *CERKL*, *NEUROD1*, *ITPRID2*, *FRZB*, *AGR3*, *LOC113071285*, *KRT18*, *klhl41a*, and *LRP2* (Figure 6a). To understand which DEGs could be more upstream regulators to others, their interaction network and gene prioritization were predicted (Figure 6b). As a result, *NEUROD1*, *FRZB*, *KRT18*, and *LRP2* could play more central roles rather than *ITPRID2* and *AGR3*. *CERKL* is predicted to be independent from the network of Figure 6b, while *LOC113071285* and *klhl41a* were not included in the database of GeneMANIA.

Among the 22 candidate genes putatively affected by coding mutations or candidate SVs, 4 of them are DEGs: *CERKL*, *ITPRID2*, *FRZB*, and *LRP2*. None of them were adjacent to the SVs. Since the three NE goldfish used for RNA-seq came from a different population than our mapping populations, we checked the BAM files for the three RNA-seq data to genotype those putative functional mutations. We found that, for the two nonsynonymous mutations of *CERKL* (chr9: 26,383,105 and 26,383,771), all the NE samples were heterozygotes. No reads were found for the other two nonsynonymous mutations of *CERKL* (chr9: 26,413,943 and 26,426,046) in the NE samples. For *ITPRID2*, heterozygotes were found in one of the NE samples for the two frameshift mutations (chr9: 26,464,773 and 26,464,774) and in all the NE samples for the nonsynonymous mutations (chr9: 26,459,566). Only two reads were aligned to one of the NE samples showing the other frameshift mutation of *ITPRID2* (chr9: 26,464,066), and they were mutant alleles; the other two NE samples were unknown for this mutation since no reads were found. For the nonsynonymous mutation of *FRZB* (chr9: 26,673,148), one NE sample was a heterozygote, and the others were homozygous mutants. For the nonsynonymous mutation (chr9: 28,575,713) and the stopgain mutation (chr9: 28,575,379) of *LRP2*, the genotypes of all six RNA-seq samples (the three CE samples were also checked) matched the candidate pattern, which is homozygous mutant in the CE samples and homozygous wild-type in the NE sample. Considering that those no-call mutations could be tightly linked with other mutations in the same gene, or not functional since not expressed, *CERKL*, *ITPRID2*, and *FRZB* were excluded from candidate genes since heterozygous mutations were detected in the NE samples. Those samples came from a population that is breed true for NEs, so there should not be carriers for the CE causal mutation. At least, no other obvious functional mutations were found affecting those three genes. Thus, *LRP2* remained the only candidate gene. Additionally, we also analyzed the RNA-seq data reported by Du et al. [14]. Comparing the whole embryo between CE and NE goldfish, *LRP2* was found to be differentially expressed in the 14-somite stage (expression in NEs is 1.7-fold higher than that in CEs, *p* < 0.01) but not in the zygote or 35% OVC stages.

In summary, by analyzing the expression profiles and coding mutations of the RNA-seq data, there is abundant evidence supporting that *LRP2* and its two coding mutations (both in Exon 38) are the top candidates for CE in goldfish. Obviously, the premature stop codon of *LRP2* (chr9: 28,575,379) leading to a truncated protein (2204 amino acid residues, while wild-type has 4529 residues, see Supplementary Text S1) is more likely to be functionally important. Three-dimensional protein structure predictions were carried out by AlphaFold (https://alphafoldserver.com/ accessed on 25 October 2025) (Figure S3). The prediction indicates that the N-terminal region (residues 1–2204), which corresponds to the entire mutant protein in the CE phenotype, is relatively buried in the wild-type structure. In contrast, the molecular surface of the wild-type protein is predominantly composed of the C-terminal region (residues 2205–4529), which is entirely absent in the mutant. This substantial alteration of the protein surface strongly suggests that the truncated LRP2 is malfunctional.

### 2.8. The CE Goldfish with Different Turning Angles of Eyeballs Are Associated with chr9:28,575,379

In our mapping populations, only a proportion of the offspring were sequenced, as they were either CEs with eyeballs turned upwards at 90° or had NEs. To investigate whether the premature stop codon in Exon 38 of LRP2 is also responsible for the CE goldfish with different angles of eyeball turning, 22 of the offspring in our mapping populations with various phenotypes were selected for genotyping the SNP of chr9:28,575,379 (C > T). As a result, in the goldfish with obvious protuberant eyes, no matter what angles of eyeball turning (from 15° to 90°) or whether there existed asymmetry of the two eyeballs (e.g., a CE goldfish with one eyeball 90° turned upwards and one 60° turned), all of them were homozygotes of mutant alleles. Other goldfish with slightly protuberant eyes (no turning of eyeballs), or NEs, were all homozygotes of wild-type alleles or heterozygotes. Therefore, we propose that the premature stop codon in Exon 38 of *LRP2* (chr9:28,575,379) is responsible for the protuberant and the upward-turning eyeballs in goldfish, while the angles of eyeball turning were affected by other factors. The fact that the CE goldfish with various turning angles of eyeballs were homozygous for the CE mutation is consistent with our deduction in our mapping population: the CE homozygous offspring were one fourth of all the offspring in both mapping populations, suggesting that a single autosomal recessive gene is responsible for CEs, and the three parental fish were heterozygous for this mutation.

## 3. Discussion

In this study, an SNP was identified as the putative causal mutation for CEs in goldfish through the construction of phenotypically segregating populations, the detection of selective regions through the whole genome, comparative genomics between different populations, transcriptomic analysis, and diagnostic tests. This SNP will lead to a truncated protein of LRP2, which clarified the genetic relationship between the TE and CE goldfish. The truncated LRP2 proteins were also reported to be the molecular mechanisms of TE goldfish in nature [10,11]. This finding could explain the phenotypic similarity between TE and CE goldfish. Meanwhile, no mutation was shared between the TE and CE goldfish, showing that they evolved independently. This is consistent with the analysis of mitochondrial genomes of the TE and CE goldfish [7]. Therefore, we discovered the parallel evolution at the molecular level between the TE and CE goldfish since different mutations in the same gene cause similar phenotypes. Moreover, we have shown that not all CE goldfish were homozygous mutants at chr9:28,575,379 (in Candidate region 9 of Figure 4, indicating that most Bj and Sh individuals were homozygous mutants); therefore, there must be other causal mutation(s) for CEs and thus other parallel evolutionary event(s) that were awaiting to be identified. Some of the pure-line CE goldfish were unable to turn their eyeballs to 90° upward, were similar to the TE goldfish without turning, or were even without the protuberance of eyes; this was observed by our study and the previous study [6]. Therefore, this reported CE-associated mutation will facilitate the mapping of other mutation(s) and also the identifying of genetically CE goldfish without expressing the phenotype for exploring other factors (genetic or environmental) affecting the development of eyes.

To be more specific, the LRP2 proteins without the 3′ portions coded by exons after Intron 45, Exon 51, or Exon 73 lead to the TE phenotype [10,11], while the truncated LRP2 protein missing the 3′ portions coded by exons after Exon 38 is associated with the CE phenotype. This suggests that the translation of Exons 38 to 45 could be necessary for preventing the turning of eyeballs, while the more downstream exons are responsible for whether the eyeballs are protuberated. However, these LRP2 proteins were found in nature. The artificially edited goldfish that had only Exons 1 to 8 translated into the LRP2 protein showed protuberated but not turned eyes [11]. The relationships between those exons and the phenotypes need further investigation. These different truncated proteins provide excellent materials for understanding the detailed molecular mechanisms of LRP2.

At the cellular level, the clinical features of celestial eyeballs in goldfish have been carefully studied [5,6]. Here we can compare them with the molecular features acquired from the current study. At the same age (~90 days), the eyeballs of the CE goldfish started to protrude and the retina started to degenerate, while the retina in the TE goldfish with protruding eyes did not obviously degenerate [6], although the TE goldfish have thinner retinas [15]. This suggests that there is no causal relationship between eye expansion and retina degeneration; instead, they are both direct consequences of the CE mutation at the same time. Therefore, the two developmental processes are discussed separately.

Regarding eye expansion, it might begin with modified lipid storage and metabolism in the eyeballs of the TE goldfish by the over-expression of genes in the PPAR signaling pathway, which could transport fatty acids outside of the eyeballs [14]. It could be similar in the CE goldfish since the up-regulation of the PPAR signaling pathway and the down-regulation of fatty acid metabolisms were observed as well in the current study. Next, the altered lipid and fatty acid content in the eyeballs could be the cause of vitreous expansion and thus elevated intraocular pressure in the TE goldfish [15] and possibly in the CE goldfish. Regarding retinal degeneration, at a later age (120 days and later, when we sampled CE goldfish for transcriptomic analysis) of the CE goldfish, the retina (including pigment epithelial cells and photoreceptors) was invaded and replaced by phagocytes and glial cells [6]. This is in line with our finding that melanogenesis, phototransduction, and phagosome pathways were affected by the CE mutation.

In addition to eye expansion and retinal degeneration, we suggest that changes may also occur in the cornea of CE goldfish. Compared with the transcriptomic analysis aimed at the TE goldfish [15], our study found that inflammatory reactions including Toll-like receptor signaling pathways were exclusively activated in the CE goldfish. Together with down-regulations of keratinocyte and cornea-related processes in the CE but not TE goldfish, a possible scenario is that the corneal keratocytes in the eyeballs of CE goldfish suffered heavy injuries, which triggers inflammation and the transformation of keratocytes into extracellular matrix (ECM) components secreting myofibroblasts [16]. At the same time, fewer keratocytes means less keratan sulfate and results in failure to maintain corneal hydration [17]. Terms or pathways related to the above processes were enriched in our transcriptomic analysis (cornea development in camera-type eye, regulation of water loss via skin, ECM–receptor interaction, etc.). Alternatively, the PPAR signaling pathway could be the direct cause of inflammation and water loss [18] instead of keratocytes. The clinical characteristics of the cornea in CE goldfish will be investigated in our future studies. More importantly, the direct cause of eyeball turning in CE goldfish remains unknown.

One limitation of this study is that the use of fish from different farms prevents us from completely ruling out the potential influence of population genetic background on transcriptomic profiles. However, the large-magnitude expression changes observed here were highly specifically enriched in pathways related to eye structures and development, which aligns closely with the ocular morphological features of CEs. Thus, we are confident that these transcriptomic changes primarily reflect divergent eye morphogenesis rather than population stratification. While this work provides important initial insights into the mechanisms underlying the CE phenotype, future studies using more rigorously controlled samples from the same genetic population will help further validate these conclusions.

Although *LRP2* is the only DEG with obvious functional mutations that match the criteria of CE candidates, other DEGs should not be excluded, as SNPs in intergenic regions could also be functional. Unfortunately, the lack of a functional motif or conservation database for goldfish hinders the discovery of these functional mutations. As shown in Figure 6b, like *LRP2*, other DEGs (*FRZB*, *NEUROD1*, and *KRT18*) might also play a central role during eye development. *FRZB* (frizzled related protein, also known as *SFRP3*) is a Wnt signaling inhibitor [19] and is evolutionally conserved in vertebrates [20]. In a study of the human ophthalmic disease Age-related Macular Degeneration (AMD), *FRZB* was identified as a mechanistic player in geographic atrophy, which is a form of AMD and is characterized by patchy degeneration of the retinal pigment epithelium and photoreceptors [21]. *NEUROD1* (also known as *NEUROD*) is a basic helix–loop–helix transcription factor critical for regulating the neuronal cell cycle; *NEUROD* knockdown in zebrafish prevented photoreceptor maturation and regeneration [22]. Furthermore, *NEUROD* in zebrafish was expressed rhythmically in differentiating photoreceptors and also in adult retinas [23]. *KRT18* encodes a type I keratin that is expressed in a wide range of tissues in humans [24]. Recently, knockdown of *krt18a.1* (a duplicated *KRT18* gene) in zebrafish suggested that it contributes to the early development of ocular neural crest cells and corneal regeneration in adults [25]. Although *CERKL* and *klhl41a* were not predicted to interact with other DEGs, other studies provide clues that they may be functional during CE development. Knockdown or knockout of *CERKL* (ceramide kinase-like) in zebrafish was generated, and the degeneration of photoreceptors and the apoptosis of retinal cells were repeatedly reported [26,27,28]. While small eyes were observed in only one *CERKL* knockdown zebrafish [27], *klhl41a* (kelch-like family member 41a) was highly expressed in eyes at 1 dpf but not 2 dpf of zebrafish embryos, and knockdown zebrafish also exhibited smaller eyes along with leaner bodies and pericardial edema [29]. *ITPRID2* and *AGR3* were predicted to interact with *FRZB* and *KRT18*, respectively (Figure 6b), but the relationship between *ITPRID2* and eye development has not been reported yet. The expression of *AGR3* was found by single-cell transcriptomics in a cluster of cells that were presumably proliferating corneal epithelial cells [30]. In addition, over-expression of *AGR2* generated enlarged eyes in *Xenopus* embryos [31], but whether *AGR3* has the same effect is unknown. *LOC113071285* is an uncharacterized protein, and thus no more information was found.

Taken together, among the aforementioned DEGs, *FRZB*, *NEUROD1*, and *KRT18* were involved only in retina- or cornea-related processes, while *klhl41a* knockdown induced zebrafish with smaller eyes, but no retina degeneration was reported. Therefore, these genes are less likely to be the cause of CEs. Although retina development and eye size were modified by knockdown or knockout of *CERKL* in zebrafish, small eyes were not constantly observed. In addition, *CERKL* was up-regulated in CE goldfish (Figure 6a), suggesting that its causality to CEs needs to be further investigated. In contrast, if the same as *AGR2*, *AGR3* over-expression could enlarge eyes, but it could not be responsible for CEs in goldfish since it was down-regulated in CE eyeballs (Figure 6a).

Lastly, *LRP2* remains the top candidate, not only because of its functional mutations and differential expression but also because of its reported functions. Multiple *LRP2* knockout mice lines showed enlarged eyes and fewer retinal cells but normal intraocular pressure [32,33,34]. Mutations in human *LRP2* lead to Donnai–Barrow and Facio-oculo-acoustico-renal (DB/FOAR) syndrome, characterized by buphthalmia (protuberant eyes), high-grade myopia, etc. [35,36]. The premature stop codon of *LRP2* in zebrafish induced naturally or artificially, similar to the causal mutations identified for the TE and CE goldfish in this study, also exhibited enlarged eyes, retina degeneration, elevated intraocular pressure, and severe myopia [37,38,39]. Furthermore, signs of phagocytes in the retina of *LRP2*-deficient mice were detected [40], resembling those in the CE goldfish [6]. The generation of a single-base mutant fish model remains a critical future step to definitively validate the causal relationship between this genotype and the CE phenotype.

## 4. Materials and Methods

### 4.1. Ethics Statement

All experiments were conducted in accordance with the Guidelines for Experimental Animals established by the Ministry of Science and Technology (Beijing, China). Animal experiments were approved by The Science Ethics Review Committee of the Beijing Academy of Agriculture and Forestry Sciences (Beijing, China) (approval number: Baafs20240901).

### 4.2. Animals and Tissue Collections

Standard Egg-fish goldfish with CEs [4] were sourced from a private keeper in Zhangjiakou, China. A mapping population was established in 2019 by crossing two female and one male of those CE goldfish. From a total of 1277 F1 offspring, 3 individuals exhibiting the NE phenotype (designated NEM, NEF1, and NEF2) were identified and selected to form the mapping population; all other offspring displayed either the CE or TE phenotype. Then, the two crosses, NEM × NEF1 and NEM × NEF2, were executed. Cross1 (NEM × NEF1) produced 309 goldfish, while 634 goldfish were obtained from Cross2 (NEM × NEF2). In Cross1, 59 goldfish with the standard CE phenotype (the eyeballs were 90° turned upwards) and 80 goldfish with the NE phenotype were collected for their tail fins; in Cross2, 64 standard CE goldfish and 80 NE goldfish were also collected for their tail fins. Tail fins from the three parental goldfish (NEM, NEF1, and NEF2) were also collected. Those goldfish were kept in a glass aquarium measuring 1.2 m in length, 0.6 m in width, and 0.45 m in height. The water was static and 35 cm deep, using groundwater that had been aerated for more than 48 h. The aquarium was indoors but experienced natural temperatures and lighting throughout the year. During the experiments, the amount of feeding was adjusted based on the body conditions of the fish to keep them healthy. A DR900 Multiparameter Portable Colorimeter (Hach, Loveland, CO, USA) was used to monitor water conditions, ensuring that pH values were between 7.0 and 8.4, dissolved oxygen ranged from 9.70 to 7.70 mg/L, nitrite was less than 0.02 mg/L, and ammonia nitrogen was less than 0.15 mg/L. The water was changed frequently depending on quality conditions. Phenotypes of the eyes of these goldfish were recorded after 12 months of age.

Eyeball tissues (without eyelids or surrounding connective tissues) for RNA-seq were collected from 3 NE goldfish from a fish farm in Jiangsu Province, China, and 3 CE goldfish from a fish farm in Beijing, China. These 6 goldfish were purchased at 6 months of age then kept in the same condition as described above for 8 months before euthanizing and sampling. Tail fins of these 6 goldfish were also collected for DNA extraction.

In addition, tail fins of CE goldfish were collected from five independent fish farms located in different regions of China, i.e., Anhui (Ah), Beijing (Bj), Hebei (Hb), Jiangsu (Js), and Shanghai (Sh) (*n* = 10 for each farm), and 10 NE goldfish from Jiangsu Province for diagnostic tests.

### 4.3. DNA Extraction and Sequencing

DNA was extracted from the aforementioned 356 fin samples using a modified CTAB method [41]. Invitrogen Qubit 4.0 (Thermo) and 0.8% agarose gel were applied for the quality control of DNA. Four DNA pools were constructed according to the shared phenotypes or crosses (Pool_CE1, Pool_NE1, Pool_CE2, and Pool_NE2). Each sample contributed 15 ng of DNA to the pool. For WGS, the 4 DNA pools and 3 parental goldfish DNA samples were prepared by a TruSeq DNA PCR-free prep kit (Illumina, San Diego, CA, USA), which built the libraries with the insertion size of ~450 bp; this was followed by the quality control accomplished by a 2100 Bioanalyzer (Agilent Technologies, Santa Clara, CA, USA) via a High Sensitivity DNA Kit (Agilent Technologies). After quantification of the libraries by QuantiFluor (Promega, Madison, WI, USA) via a Quant-iT PicoGreen dsDNA Assay Kit (Thermo Fisher Scientific, Waltham, MA, USA), the qualified libraries were 2 × 150 bp paired-end sequenced by Illumina NovaSeq with 30X coverage (the 4 offspring pools) or 10X coverage (the 3 parental samples). The 50 CE goldfish samples were also prepared and sequenced individually with 4X coverage. All DNA extraction and sequencing were carried out by Personalbio Technology Co., Ltd. (Shanghai, China), and the raw data was deposited in the Genome Sequence Archive [42] in the National Genomics Data Center (NGDC) [43], China National Center for Bioinformation/Beijing Institute of Genomics, Chinese Academy of Sciences.

### 4.4. Alignment of WGS Data and Calling of SNPs, Indels, and SVs

Raw sequencing data were analyzed and quality-controlled using FastQC (version 0.12.1) [44]. Then, the clean data were aligned to the goldfish reference genome [12]. All FASTQ clean data were aligned to the reference genome using BWA-MEM (version: 0.7.12-r1039) [45] with default parameters. Then, the SAM files were sorted and converted to BAM files by SAMtools (version: 1.17) [46].

After alignment, SNPs and Indels were called with GATK HaplotypeCaller 3.8 [47], but no filtration was applied to avoid excluding possible causal mutations, and they were annotated by ANNOVAR (version 2019-10-24) [48]. Structural variants were called with Lumpy (version: 0.2.13) [49].

### 4.5. Diagnostic Test for the Reported TE Causal Mutations

PCR protocols with a three-primer system were designed to genotype the ~13 kb insertion in the 45th intron of the *lrp2aL* gene [10]. The primers are LRP2_Exon45_F (5′-GCAGTGATGGTTCGGATGAG-3′), LRP2_Exon46_R (5′-AACTGGTCGGAGTTGCAGGT-3′), and gFV-1_R (5′-CCCAGTGAGACACGATTGGA-3′, or gFV-1_F: 5′-AGATTGCCTTTGCTGGTTTGA-3′). LRP2_Exon45_F and LRP2_Exon46_R can amplify a 1710 bp fragment when the 13 kb insertion is absent, while LRP2_Exon45_F and gFV-1_R (or LRP2_Exon46_R and gFV-1_F) can only amplify fragments when the allele with the ~13 kb insertion exists (since the exact insertion site is unknown, the precise PCR product sizes are not clear but should be less than 1710 bp). In addition, a two-primer system PCR was also carried out using primers LRP2_Exon45_F and LRP2_Exon46_R for Sanger sequencing of Intron 45 of LRP2. Samples used for sequencing the 45th Intron of the LRP2 gene were 2 randomly selected CE goldfish in the offspring of Cross1 and another 2 random CE fish in the offspring of Cross2.

The three-primer PCR systems included 10 μL of 2 × Tap Plus PCR MasterMix (Solarbio, Beijing, China), 0.5 μL of each primer (10 μM concentration), 1 μL of genomic DNA (50 ng/μL concentration), and ddH_2_O added up to 20 μL. This protocol was used for all the PCR runs of 94 °C for 3 min, 30 cycles of 94 °C for 30 s, 60 °C for 30 s, and 72 °C for 1 min each, followed by 72 °C for 10 min. Amplifications were carried out on a Veriti 96 well thermal cycler (Applied Biosystems, Thermo, Waltham, MA, USA). PCR products were assessed by agarose gel electrophoresis or Sanger sequencing (Tsingke Biotechnology, Beijing, China).

### 4.6. Identification of Candidate Regions and Mutations

The BAM files for Pool_CE1 and Pool_NE1 were used to calculate pair-wise F_ST_ values with Popoolation2 [50] with a sliding window approach and with a window size of 50 kb and step size of 10 kb. Allele frequency differences (dAFs) between the 2 pools were also calculated by Popoolation2. Pool_CE2 and Pool_NE2 were analyzed according to the same procedure. The F_ST_ values were Z-transformed, and genomic regions with ZF_ST_ values higher than 11 were defined as candidate regions (the sum of high ZF_ST_ regions detected in Cross1 and Cross2). Within the candidate regions, variants with dAFs larger than 0.5 while being heterozygous in both the parents were considered as candidate mutations. Next, all candidate mutations that were fixed in the 50 individual WGS data from 5 different fish farms were screened out.

For each of the candidate regions, the pair-wise genetic distances between the 52 CE goldfish libraries (2 pools and 50 individuals) were evaluated by the following steps: 1. The QC of the raw output of GATK HaplotypeCaller consisted of “QD < 2.0 || FS > 60.0 || MQ < 40.0 || MQRankSum < −12.5 || ReadPosRankSum < −8.0” of the filter-expression option in GATK VariantFiltration for SNPs, while Indels were excluded; 2. The filtered SNPs were phased by beagle (version: 5.1) [51]; 3. Python script distMat.py (version: 0.4 https://github.com/simonhmartin/genomics_general, accessed on 15 January 2024) was applied to calculate the pair-wise genetic distances between all the 52 libraries. Any of the 50 individual samples showing a genetic distance from Pool_CE (1 or 2) larger than 0.1 were excluded from the screening of target mutations for that region, since these sequences were considered to have originated differently from our mapping population and thus to not be sharing the same causal mutation.

All the SVs within or flanking the target region (1 Mb upstream and 1 Mb downstream), plus candidate coding SNPs and Indels, were selected for further analysis. All the selected SVs were double checked for their reliability and allelic frequencies in the 7 libraries from the mapping populations by viewing the BAM file to sift out the ones that were reliable and also matched the criteria “heterogeneous in the 3 parental samples and 2 NE pools but homozygous in the 2 CE pools”.

### 4.7. RNA Extraction and Transcriptomic Analysis

For the RNA-seq analysis, total RNA was extracted from the three NE and three CE goldfish using Trizol (Invitrogen, Carlsbad, CA, USA) according to the manufacturer’s protocol. Library products were prepared, and the NovaSeq 6000 platform (Illumina) was applied by Personalbio Technology Co., Ltd. (Shanghai, China). Cutadapt (version: 1.15) [52], Hisat2 (version: 2.0.5) [53], and HTseq (version: 0.9.1) [54] software were used for quality control, aligning to the goldfish reference genome [12] and counting for the read number for each gene. Next, DESeq (version: 1.30.0) [55] was applied for detecting differentially expressed genes between the NE and CE groups. The criteria for DEGs are |log2foldchange| > 1 and adjusted *p*-value < 0.05. Then, GO and KEGG enrichment analyses for the DEGs were conducted by the clusterProfiler package (version: 3.4.4) of R [56]. The same protocols were applied to all the raw short-read RNA-seq data uploaded by Du et al. [13] (PRJNA558211).

Interaction networks and gene prioritization for candidate genes were predicted using the online platform GeneMANIA (https://genemania.org, accessed on 21 February 2024) [57].

### 4.8. Diagnostic Test for the Candidate Mutation for CE

To genotype the premature stop codon in *LRP2* (chr9: 28,575,379), primers were designed: LRP2_Exon38_F (5′-GGACCACCGAGACGGATACA-3′) and LRP2_Exon38_R (5′-TGGGATGGCGAAGCAGA-3′). The amplicon is 361 bp and the same PCR protocol was applied as mentioned above. Both forward and reverse primers were used for Sanger sequencing for double checking. The Sanger-sequenced goldfish were CE offspring from our mapping population (*n* = 11), including individuals with eyeballs turned upwards at 15° (*n* = 1), 30° (*n* = 2), 45° (*n* = 1), 60° (*n* = 2), 70° (*n* = 1), one eye 80° and another eye 60° (*n* = 1), one eye 90° and another eye 60° (*n* = 1), and one eye 90° and another eye 70° (*n* = 2). In addition, the offspring with slightly protuberant eyes (no turning of eyeballs, *n* = 5) and NEs (*n* = 6) were also selected for Sanger sequencing.

### 4.9. Statistical Analysis

For FPKM, comparisons between two groups were performed using a two-sided Student’s *t*-test. A *p*-value < 0.05 was considered significant. All data are presented as means ± SEM.

## 5. Conclusions

The gene mapping in this study suggests that coding mutations in the *LRP2* gene are responsible for CEs in goldfish, which expands our knowledge regarding the genetics and evolution of TEs and CEs at the molecular level. Moreover, transcriptomic analysis provides clues regarding the cellular processes contributing to the CE phenotypes.

## Data Availability

The raw RNA sequencing reads generated in this study have been submitted to NCBI (PRJNA1161404) and can be found at https://www.ncbi.nlm.nih.gov/bioproject/PRJNA1161404, accessed on 15 September 2024. The raw WGS sequence data reported in this paper have been deposited in the Genome Sequence Archive in NGDC (GSA: CRA021140), which are publicly accessible at https://ngdc.cncb.ac.cn/gsa/browse/CRA021140, accessed on 14 December 2024. All the shell scripts involved in this study are included in Supplementary Text S2.

## Supplementary Materials

The following supporting information can be downloaded at https://www.mdpi.com/article/10.3390/ijms262110625/s1.

## Abbreviations

The following abbreviations are used in this manuscript:

CE	Celestial eye
TE	Telescope eye
NE	Normal eye
NEM	Normal eye male
NEF	Normal eye female
ZF_ST_	Z-Score normalized fixation index
dAF	Allelic frequency differences
SV	Structural variance
WT	Wild-type
IBD	Identity-by-descent
DEG	Differentially expressed genes
GO	Gene Ontology
KEGG	Kyoto Encyclopedia of Genes and Genomes
PPAR	Peroxisome proliferator-activated receptor
OVC	Otic vesicle closure
ECM	Extracellular matrix
AMD	Age-related Macular Degeneration
DB/FOAR	Donnai–Barrow and Facio-oculo-acoustico-renal
Ah	Anhui
Bj	Beijing
Hb	Hebei
Js	Jiangsu
Sh	Shanghai
CTAB	Cetyltrimethylammonium Bromide
NGDC	National Genomics Data Center

## References

[1] Wang S.Y., Luo J., Murphy R.W., Wu S.F., Zhu C.L., Gao Y., Zhang Y.P. (2013). Origin of Chinese goldfish and sequential loss of genetic diversity accompanies new breeds. PLoS ONE.

[2] Ota K.G., Abe G. (2016). Goldfish morphology as a model for evolutionary developmental biology. Wiley Interdiscip. Rev. Dev. Biol..

[3] Le Verger K., Küng L.C., Fabre A.-C., Schmelzle T., Wegmann A., Sánchez-Villagra M.R. (2024). Goldfish phenomics reveals commonalities and a lack of universality in the domestication process for ornamentation. Evol. Lett..

[4] Wang H. (2000). Atlas of Chinese Goldfish.

[5] Matsumura M., Ohkuma M., Honda Y. (1981). Electron microscopic studies on celestial goldfish retina—A possible new type of retinal degeneration in experimental animals. Exp. Eye Res..

[6] Matsumura M., Ohkuma M., Honda Y. (1982). Retinal degeneration in celestial goldfish: Developmental study. Ophthalmic Res..

[7] Komiyama T., Kobayashi H., Tateno Y., Inoko H., Gojobori T., Ikeo K. (2009). An evolutionary origin and selection process of goldfish. Gene.

[8] Smartt J. (2001). Goldfish Varieties and Genetics: A Handbook for Breeders.

[9] Omori Y., Kon T. (2019). Goldfish: An old and new model system to study vertebrate development, evolution and human disease. J. Biochem..

[10] Kon T., Omori Y., Fukuta K., Wada H., Watanabe M., Chen Z., Iwasaki M., Mishina T., Matsuzaki S.-I.S., Yoshihara D. (2020). The genetic basis of morphological diversity in domesticated goldfish. Curr. Biol..

[11] Yu P., Wang Y., Li Z., Jin H., Li L.-L., Han X., Wang Z.-W., Yang X.-L., Li X.-Y., Zhang X.-J. (2022). Causal gene identification and desirable trait recreation in goldfish. Sci. China Life Sci..

[12] Chen D., Zhang Q., Tang W., Huang Z., Wang G., Wang Y., Shi J., Xu H., Lin L., Li Z. (2020). The evolutionary origin and domestication history of goldfish (*Carassius auratus*). Proc. Natl. Acad. Sci. USA.

[13] Mirimin L., Roodt-Wilding R. (2015). Testing and validating a modified CTAB DNA extraction method to enable molecular parentage analysis of fertilized eggs and larvae of an emerging South African aquaculture species, the dusky kob *Argyrosomus japonicus*. J. Fish Biol..

[14] Chen T., Chen X., Zhang S., Zhu J., Tang B., Wang A., Dong L., Zhang Z., Yu C., Sun Y. (2021). The genome sequence archive family: Toward explosive data growth and diverse data types. Genom. Proteom. Bioinform..

[15] Members C.-N., Partners C.-N.M.A., Xue Y., Bao Y., Zhao W., Xiao J., He S., Zhang G., Li Y., Zhao G. (2022). Database Resources of the National Genomics Data Center, China National Center for Bioinformation in 2022. Nucleic Acids Res..

[16] Li H. (2013). Aligning sequence reads, clone sequences and assembly contigs with BWA-MEM. arXiv.

[17] Li H., Handsaker B., Wysoker A., Fennell T., Ruan J., Homer N. (2009). The sequence alignment/map format and SAMtools. Bioinformatics.

[18] Wang K., Li M., Hakonarson H. (2010). ANNOVAR: Functional annotation of genetic variants from high-throughput sequencing data. Nucleic Acids Res..

[19] Layer R.M., Chiang C., Quinlan A.R., Hall I.M. (2014). LUMPY: A probabilistic framework for structural variant discovery. Genome Biol..

[20] Martin M. (2011). Cutadapt removes adapter sequences from high-throughput sequencing reads. EMBnet J..

[21] Kim D., Paggi J.M., Park C., Bennett C., Salzberg S.L. (2019). Graph-based genome alignment and genotyping with HISAT2 and HISAT-genotype. Nat. Biotechnol..

[22] Du X., Zhang W., Wu J., You C., Dong X. (2023). Full-length RNA sequencing provides insights into goldfish evolution under artificial selection. Int. J. Mol. Sci..

[23] Warde-Farley D., Donaldson S.L., Comes O., Zuberi K., Badrawi R., Chao P., Franz M., Grouios C., Kazi F., Lopes C.T. (2010). The GeneMANIA prediction server: Biological network integration for gene prioritization and predicting gene function. Nucleic Acids Res..

[24] Yu P., Wang Y., Yang W.-T., Li Z., Zhang X.-J., Zhou L., Gui J.-F. (2021). Upregulation of the PPAR signaling pathway and accumulation of lipids are related to the morphological and structural transformation of the dragon-eye goldfish eye. Sci. China Life Sci..

[25] Raymond P., Spilman D., Hill R., Bahn C. (1984). The telescopic eyes of Black Moor goldfish: Elevated intraocular pressure and altered aqueous outflow pathways. Investig. Ophthalmol. Vis. Sci..

[26] Wilson S.E., Chaurasia S.S., Medeiros F.W. (2007). Apoptosis in the initiation, modulation and termination of the corneal wound healing response. Exp. Eye Res..

[27] Funderburgh J.L. (2000). MINI REVIEW Keratan sulfate: Structure, biosynthesis, and function. Glycobiology.

[28] Wang J., Chen S., Zhao X., Guo Q., Yang R., Zhang C., Huang Y., Ma L., Zhao S. (2023). Effect of PPARγ on oxidative stress in diabetes-related dry eye. Exp. Eye Res..

[29] Bennett C.N., Ross S.E., Longo K.A., Bajnok L., Hemati N., Johnson K.W., Harrison S.D., MacDougald O.A. (2002). Regulation of Wnt signaling during adipogenesis. J. Biol. Chem..

[30] Oh C.-K., Ko Y., Park J.J., Heo H.J., Kang J., Kwon E.J., Lee Y., Myung K., Kang J.M., Ko D.S. (2021). FRZB as a key molecule in abdominal aortic aneurysm progression affecting vascular integrity. Biosci. Rep..

[31] Orozco L.D., Owen L.A., Hofmann J., Stockwell A.D., Tao J., Haller S., Mukundan V.T., Clarke C., Lund J., Sridhar A. (2023). A systems biology approach uncovers novel disease mechanisms in age-related macular degeneration. Cell Genom..

[32] Taylor S.M., Alvarez-Delfin K., Saade C.J., Thomas J.L., Thummel R., Fadool J.M., Hitchcock P.F. (2015). The bHLH transcription factor NeuroD governs photoreceptor genesis and regeneration through delta-notch signaling. Investig. Ophthalmol. Vis. Sci..

[33] Laranjeiro R., Whitmore D. (2014). Transcription factors involved in retinogenesis are co-opted by the circadian clock following photoreceptor differentiation. Development.

[34] Ho M., Thompson B., Fisk J.N., Nebert D.W., Bruford E.A., Vasiliou V., Bunick C.G. (2022). Update of the keratin gene family: Evolution, tissue-specific expression patterns, and relevance to clinical disorders. Hum. Genom..

[35] Williams A.L., Bohnsack B.L. (2024). Keratin 8/18a.1 expression influences embryonic neural crest cell dynamics and contributes to postnatal corneal regeneration in zebrafish. Cells.

[36] Yu S., Li C., Biswas L., Hu X., Liu F., Reilly J., Liu X., Liu Y., Huang Y., Lu Z. (2017). CERKL gene knockout disturbs photoreceptor outer segment phagocytosis and causes rod-cone dystrophy in zebrafish. Hum. Mol. Genet..

[37] Riera M., Burguera D., Garcia-Fernàndez J., Gonzàlez-Duarte R. (2013). CERKL knockdown causes retinal degeneration in zebrafish. PLoS ONE.

[38] Li C., Wang L., Zhang J., Huang M., Wong F., Liu X., Liu F., Cui X., Yang G., Chen J. (2014). CERKL interacts with mitochondrial TRX2 and protects retinal cells from oxidative stress-induced apoptosis. Biochim. Biophys. Acta.

[39] Gupta V.A., Ravenscroft G., Shaheen R., Todd E.J., Swanson L.C., Shiina M., Ogata K., Hsu C., Clarke N.F., Darras B.T. (2013). Identification of KLHL41 mutations implicates BTB-Kelch-mediated ubiquitination as an alternate pathway to myofibrillar disruption in nemaline myopathy. Am. J. Hum. Genet..

[40] Howard L., Ishikawa Y., Katayama T., Park S.-J., Hill M.J., Blake D.J., Nishida K., Hayashi R., Quantock A.J. (2024). Single-cell transcriptomics reveals the molecular basis of human iPS cell differentiation into ectodermal ocular lineages. Commun. Biol..

[41] Tereshina M.B., Ivanova A.S., Eroshkin F.M., Korotkova D.D., Nesterenko A.M., Bayramov A.V., Solovieva E.A., Parshina E.A., Orlov E.E., Martynova N.Y. (2019). Agr2-interacting Prod1-like protein Tfp4 from *Xenopus laevis* is necessary for early forebrain and eye development as well as for the tadpole appendage regeneration. Genesis.

[42] Cases O., Joseph A., Obry A., Santin M.D., Ben-Yacoub S., Pâques M., Amsellem-Levera S., Bribian A., Simonutti M., Augustin S. (2015). Foxg1-Cre mediated Lrp2 inactivation in the developing mouse neural retina, ciliary and retinal pigment epithelia models congenital high myopia. PLoS ONE.

[43] Mai S., Zhu X., Wan E.Y.C., Wu S., Yonathan J.N., Wang J., Li Y., Ma J.Y.W., Zuo B., Tse D.Y.-Y. (2022). Postnatal eye size in mice is controlled by SREBP2-mediated transcriptional repression of Lrp2 and Bmp2. Development.

[44] Chen S., Zhou Y., Chen Y., Gu J. (2018). fastp: An ultra-fast all-in-one FASTQ preprocessor. Bioinformatics.

[45] Storm T., Burgoyne T., Dunaief J.L., Christensen E.I., Futter C., Nielsen R. (2019). Selective ablation of megalin in the retinal pigment epithelium results in megaophthalmos, macromelanosome formation and severe retina degeneration. Investig. Ophthalmol. Vis. Sci..

[46] Pober B.R., Longoni M., Noonan K.M. (2009). A review of Donnai-Barrow and facio-oculo-acoustico-renal (DB/FOAR) syndrome: Clinical features and differential diagnosis. Birth Defects Res. A Clin. Mol. Teratol..

[47] McKenna A., Hanna M., Banks E., Sivachenko A., Cibulskis K., Kernytsky A., Garimella K., Altshuler D., Gabriel S., Daly M. (2010). The Genome Analysis Toolkit: A MapReduce framework for analyzing next-generation DNA sequencing data. Genome Res..

[48] Kantarci S., Al-Gazali L., Hill R.S., Donnai D., Black G.C.M., Bieth E., Chassaing N., Lacombe D., Devriendt K., Teebi A. (2007). Mutations in LRP2, which encodes the multiligand receptor megalin, cause Donnai-Barrow and facio-oculo-acoustico-renal syndromes. Nat. Genet..

[49] Collery R.F., Link B.A. (2019). Precise short sequence insertion in zebrafish using a CRISPR/Cas9 approach to generate a constitutively soluble Lrp2 protein. Front. Cell Dev. Biol..

[50] Kofler R., Pandey R.V., Schlötterer C. (2011). PoPoolation2: Identifying differentiation between populations using sequencing of pooled DNA samples (Pool-Seq). Bioinformatics.

[51] Browning S.R., Browning B.L. (2007). Rapid and accurate haplotype phasing and missing-data inference for whole-genome association studies by use of localized haplotype clustering. Am. J. Hum. Genet..

[52] Sherpa T., Hunter S.S., Frey R.A., Robison B.D., Stenkamp D.L. (2011). Retinal proliferation response in the buphthalmic zebrafish, bugeye. Exp. Eye Res..

[53] Veth K.N., Willer J.R., Collery R.F., Gray M.P., Willer G.B., Wagner D.S., Mullins M.C., Udvadia A.J., Smith R.S., John S.W.M. (2011). Mutations in zebrafish lrp2 result in adult-onset ocular pathogenesis that models myopia and other risk factors for glaucoma. PLoS Genet..

[54] Anders S., Pyl P.T., Huber W. (2015). HTSeq—A Python framework to work with high-throughput sequencing data. Bioinformatics.

[55] Anders S., Huber W. (2010). Differential expression analysis for sequence count data. Nat. Preced..

[56] Yu G., Wang L.G., Han Y., He Q.Y. (2012). clusterProfiler: An R package for comparing biological themes among gene clusters. Omics J. Integr. Biol..

[57] Cases O., Obry A., Ben-Yacoub S., Augustin S., Joseph A., Toutirais G., Simonutti M., Christ A., Cosette P., Kozyraki R. (2017). Impaired vitreous composition and retinal pigment epithelium function in the FoxG1: LRP2 myopic mice. Biochim. Biophys. Acta.

