# Supplementary material for: Independent Mutations in the LRP2 Gene Mediating Telescope Eyes and Celestial Eyes in Goldfish
Source: Int J Mol Sci. 2025 Oct 31;26(21):10625. doi: 10.3390/ijms262110625 (PMC12608239; doi:10.3390/ijms262110625)
Supplement: Supplementary file 1 [file ijms-26-10625-s001.zip › ijms-3914209-supplementary.pdf]

SUPPLEMENTAL MATERIAL

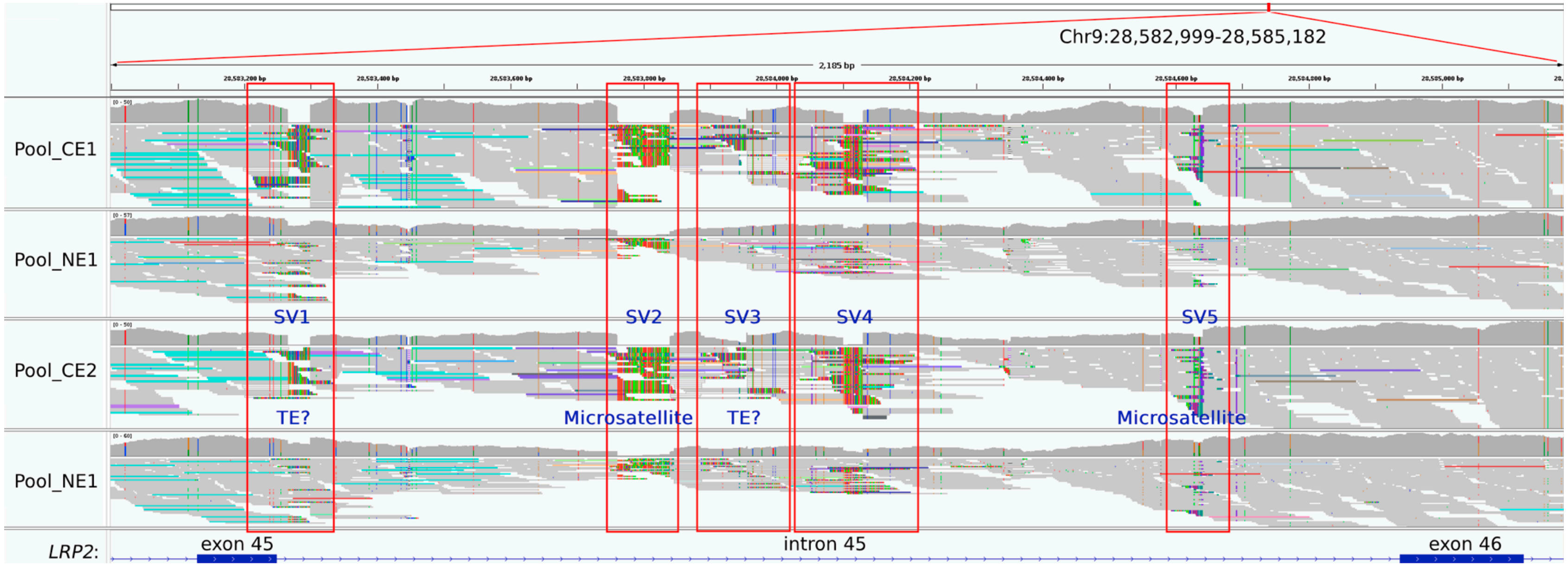

Figure S1. The SVs detected in the intron 45 of *LRP2* gene. “TE?” in this figure indicating that these SVs (SV1 and SV3) could be transposon elements because of the surrounding reads (mates mapped in a different chromosome, and secondary mapping). The allelic frequencies of these 5 SVs matched the expected pattern for causal mutations of CE except SV2 which is almost 100% in 4 DNA pools. Sanger sequencing has confirmed that SV1 is a 33 bp sequence (chr9:28,583,267-28,583,299) replaced by a 116 bp sequence, and also the previously reported ~13 kb insertion is absent

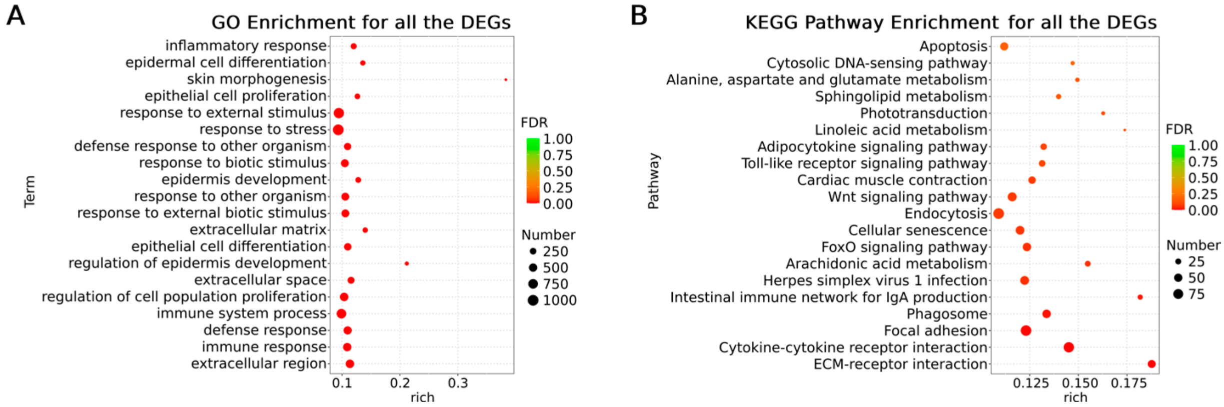

Figure S2. Enrichment analysis of the differentially expressed genes (DEGs) between eyeballs of NE and CE goldfish via RNA-seq. Top 20 GO terms (*A*) and top 20 KEGG pathways (*B*) significantly enriched for all the DEGs. In each of *A* or *B*, the *P*-value increases as the terms or pathways are presented from the bottom to the top.

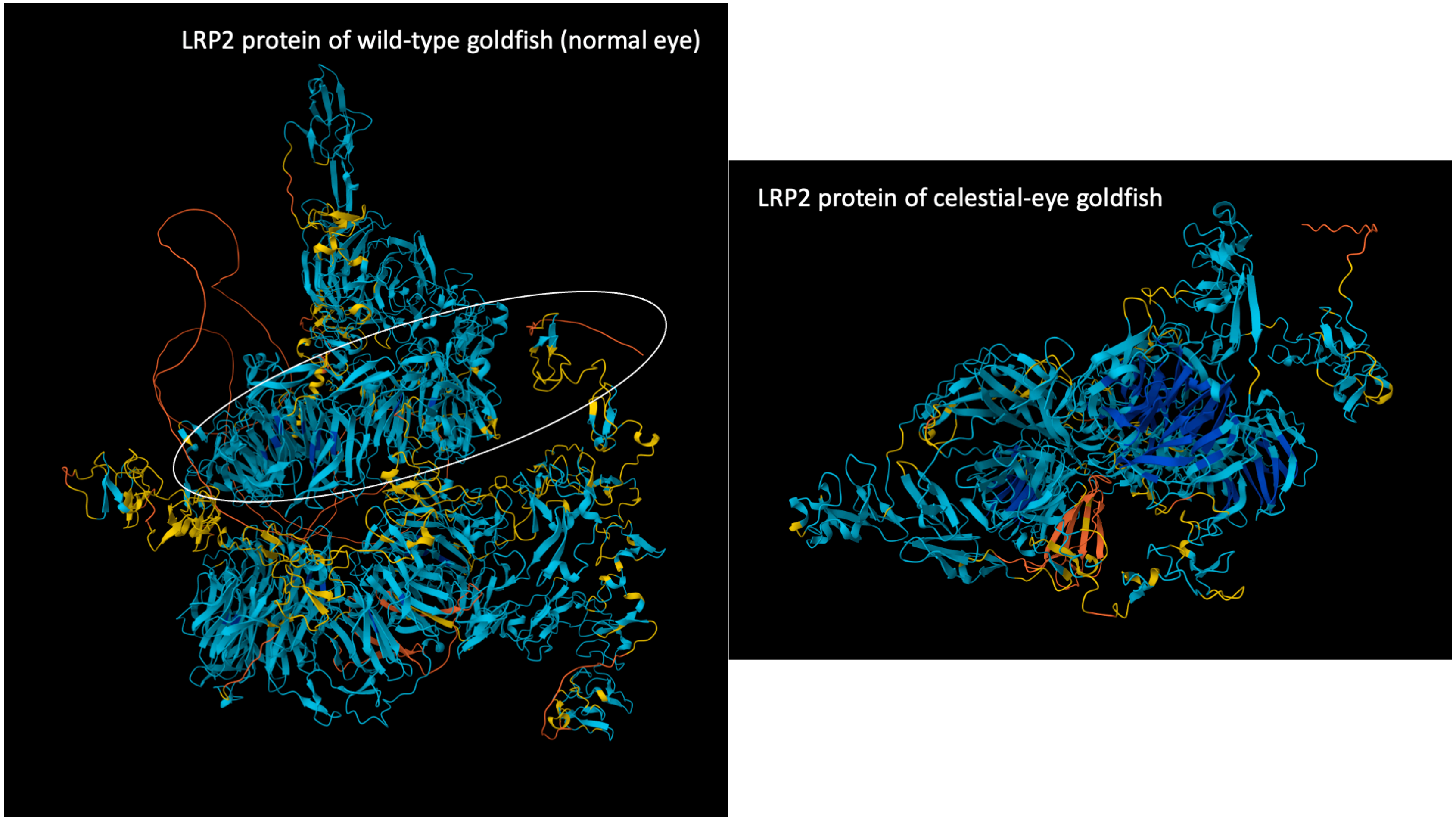

Figure S3. Predicted three-dimensional structures of wild-type and mutant LRP2 in goldfish. Structures were generated using AlphaFold. The wild-type LRP2 protein (4,529 residues) is shown on the left. The N-terminal region (residues 1-2,204), which constitutes the entirety of the mutant protein, is located in a central, buried position (indicated by the white circle). The truncated LRP2 mutant protein (2,204 residues) is shown on the right, resulting from the premature stop codon identified in this study (chr9:28,575,379). The color spectrum from blue to orange indicates the per-residue model confidence, with blue representing high confidence and yellow/orange representing lower confidence.

Table S1. Candidate regions of CE in goldfish through the comparisons of offspring in mapping populations by pooled WGS. Candidate regions were defined as ZF_{ST} larger than 11. Firstly, candidate regions in Cross1 and Cross2 were defined separately (middle and right panel in the table, respectively), then they were combined into the candidate regions in the left panel in the table.

<table>
<tr><th colspan="4">Cross1 and 2 combined (9 candidate regions)</th><th colspan="4">Cross1 (7 candidate regions)</th><th colspan="4">Cross2 (8 candidate regions)</th></tr>
<tr><th>Name</th><th>Start (Mb)</th><th>End (Mb)</th><th>Size (Mb)</th><th>Start (Mb)</th><th>End (Mb)</th><th>Size (Mb)</th><th>ZF_{ST}</th><th>Start (Mb)</th><th>End (Mb)</th><th>Size (Mb)</th><th>ZF_{ST}</th></tr>
<tr><td>Candidate region 1</td><td>19.73</td><td>19.79</td><td>0.06</td><td></td><td></td><td></td><td></td><td>19.73</td><td>19.79</td><td>0.06</td><td>16.83</td></tr>
<tr><td>Candidate region 2</td><td>19.99</td><td>20.04</td><td>0.05</td><td></td><td></td><td></td><td></td><td>19.99</td><td>20.04</td><td>0.05</td><td>17.01</td></tr>
<tr><td>Candidate region 3</td><td>21.39</td><td>21.57</td><td>0.18</td><td>21.39</td><td>21.57</td><td>0.18</td><td>12.55</td><td>21.4</td><td>21.57</td><td>0.17</td><td>12.23</td></tr>
<tr><td rowspan="2">Candidate region 4</td><td rowspan="2">21.64</td><td rowspan="2">22.01</td><td rowspan="2">0.37</td><td>21.64</td><td>21.92</td><td>0.28</td><td>13.14</td><td rowspan="2">21.64</td><td rowspan="2">22</td><td rowspan="2">0.36</td><td rowspan="2">12.04</td></tr>
<tr><td>21.95</td><td>22.01</td><td>0.06</td><td>11.71</td></tr>
<tr><td>Candidate region 5</td><td>23.5</td><td>23.55</td><td>0.05</td><td>23.5</td><td>23.55</td><td>0.05</td><td>11.08</td><td></td><td></td><td></td><td></td></tr>
<tr><td>Candidate region 6</td><td>24.38</td><td>24.43</td><td>0.05</td><td></td><td></td><td></td><td></td><td>24.38</td><td>24.43</td><td>0.05</td><td>11.19</td></tr>
<tr><td>Candidate region 7</td><td>25.3</td><td>26.77</td><td>1.47</td><td>25.31</td><td>26.77</td><td>1.46</td><td>15.98</td><td>25.3</td><td>26.77</td><td>1.47</td><td>17.77</td></tr>
<tr><td>Candidate region 8</td><td>26.83</td><td>27.62</td><td>0.79</td><td>26.83</td><td>27.62</td><td>0.79</td><td>17.87</td><td>26.83</td><td>27.62</td><td>0.79</td><td>18.18</td></tr>
<tr><td>Candidate region 9</td><td>28.53</td><td>28.63</td><td>0.1</td><td>28.53</td><td>28.63</td><td>0.1</td><td>19.32</td><td>28.53</td><td>28.63</td><td>0.1</td><td>19.19</td></tr>
</table>

*The coordinates refer to the chromosome 9 in goldfish genome assembly reported by Chen et al., in 2020.

Table S2. The identity regions shared by the 50 CE goldfish from 5 different populations. The data is coordinated with Fig. 4, showing that no region was shared by all the samples.

Samples	Candidate region 1	Candidate region 2	Candidate region 3	Candidate region 4	Candidate region 5	Candidate region 6	Candidate region 7	Candidate region 8	Candidate region 9
Ah_1	IBD	IBD	Excluded	Excluded	Excluded	Excluded	Excluded	Excluded	Excluded
Ah_2	IBD	IBD	Excluded	Excluded	Excluded	Excluded	Excluded	Excluded	Excluded
Ah_3	Excluded	Excluded	Excluded	Excluded	Excluded	Excluded	Excluded	Excluded	Excluded
Ah_4	IBD	IBD	Excluded	Excluded	Excluded	Excluded	Excluded	Excluded	Excluded
Ah_5	IBD	IBD	Excluded	Excluded	Excluded	Excluded	Excluded	Excluded	Excluded
Ah_6	IBD	IBD	Excluded	Excluded	Excluded	Excluded	Excluded	Excluded	Excluded
Ah_7	Excluded	Excluded	Excluded	Excluded	Excluded	Excluded	Excluded	Excluded	Excluded
Ah_8	IBD	IBD	Excluded	Excluded	Excluded	IBD	Excluded	Excluded	Excluded
Ah_9	Excluded	Excluded	Excluded	Excluded	Excluded	Excluded	Excluded	Excluded	Excluded
Ah_10	IBD	IBD	Excluded	Excluded	Excluded	Excluded	Excluded	Excluded	Excluded
Bj_1	IBD	IBD	Excluded	Excluded	IBD	IBD	IBD	IBD	IBD
Bj_2	IBD	IBD	Excluded	Excluded	IBD	IBD	Excluded	Excluded	Excluded
Bj_3	IBD	IBD	Excluded	Excluded	IBD	IBD	IBD	IBD	IBD
Bj_4	IBD	IBD	Excluded	Excluded	IBD	IBD	IBD	IBD	IBD
Bj_5	IBD	IBD	Excluded	Excluded	IBD	IBD	IBD	IBD	IBD
Bj_6	IBD	IBD	Excluded	Excluded	IBD	IBD	IBD	IBD	IBD
Bj_7	IBD	IBD	Excluded	Excluded	IBD	IBD	IBD	IBD	IBD
Bj_8	IBD	IBD	Excluded	Excluded	IBD	IBD	IBD	IBD	IBD
Bj_9	IBD	IBD	Excluded	Excluded	IBD	IBD	IBD	IBD	IBD
Bj_10	IBD	IBD	Excluded	Excluded	IBD	IBD	IBD	IBD	IBD
Hb_1	IBD	IBD	Excluded	Excluded	IBD	IBD	Excluded	Excluded	Excluded
Hb_2	IBD	IBD	Excluded	Excluded	IBD	IBD	Excluded	Excluded	Excluded
Hb_3	IBD	IBD	Excluded	Excluded	IBD	IBD	Excluded	Excluded	Excluded
Hb_4	IBD	IBD	Excluded	Excluded	IBD	IBD	Excluded	Excluded	Excluded
Hb_5	IBD	IBD	Excluded	Excluded	Excluded	IBD	Excluded	Excluded	Excluded
Hb_6	IBD	IBD	Excluded	Excluded	Excluded	IBD	Excluded	Excluded	Excluded
Hb_7	IBD	IBD	Excluded	Excluded	IBD	IBD	Excluded	Excluded	Excluded
Hb_8	IBD	IBD	Excluded	Excluded	IBD	IBD	Excluded	Excluded	Excluded
Hb_9	IBD	IBD	Excluded	Excluded	IBD	IBD	Excluded	Excluded	Excluded
Hb_10	IBD	IBD	Excluded	Excluded	IBD	IBD	Excluded	Excluded	Excluded
Js_1	IBD	IBD	Excluded	Excluded	Excluded	Excluded	Excluded	IBD	Excluded
Js_2	IBD	IBD	Excluded	Excluded	Excluded	Excluded	Excluded	IBD	Excluded
Js_3	IBD	IBD	Excluded	Excluded	Excluded	Excluded	Excluded	IBD	Excluded
Js_4	IBD	IBD	Excluded	Excluded	Excluded	Excluded	Excluded	IBD	Excluded
Js_5	IBD	IBD	Excluded	Excluded	Excluded	Excluded	Excluded	IBD	Excluded

Js_6	IBD	IBD	Excluded	Excluded	Excluded	Excluded	Excluded	IBD	Excluded
Js_7	IBD	IBD	Excluded	Excluded	Excluded	Excluded	Excluded	IBD	Excluded
Js_8	IBD	IBD	Excluded	Excluded	Excluded	Excluded	Excluded	IBD	Excluded
Js_9	IBD	IBD	Excluded	Excluded	Excluded	Excluded	Excluded	IBD	Excluded
Js_10	IBD	IBD	Excluded	Excluded	Excluded	Excluded	Excluded	IBD	Excluded
Sh_1	IBD	IBD	Excluded	Excluded	Excluded	Excluded	Excluded	IBD	Excluded
Sh_2	IBD	IBD	Excluded	Excluded	Excluded	IBD	Excluded	Excluded	Excluded
Sh_3	IBD	IBD	Excluded	Excluded	Excluded	IBD	IBD	Excluded	IBD
Sh_4	IBD	IBD	Excluded	Excluded	Excluded	IBD	Excluded	Excluded	IBD
Sh_5	IBD	IBD	Excluded	Excluded	IBD	IBD	IBD	IBD	IBD
Sh_6	IBD	IBD	Excluded	Excluded	Excluded	IBD	Excluded	Excluded	Excluded
Sh_7	IBD	IBD	Excluded	Excluded	Excluded	IBD	IBD	Excluded	IBD
Sh_8	IBD	IBD	Excluded	Excluded	Excluded	IBD	IBD	Excluded	IBD
Sh_9	IBD	IBD	Excluded	Excluded	Excluded	IBD	Excluded	Excluded	Excluded
Sh_10	IBD	IBD	Excluded	Excluded	Excluded	IBD	IBD	IBD	IBD

*The 9 candidate regions refer to Supplemental Table S1.

*"IBD" means the genetic distance with Pool_CE (1 or 2) is smaller than 0.1, "Excluded" means larger than 0.1.

Supplementary Text S1: Amino acid sequences of wild-type and truncated LRP2 protein by the premature stop codon identified in this study (chr9: 28,575,379)

>Wild-type LRP2 protein (4,529 AA)
MERPTVRITQTKLAAVTQPTCESAQFQCLSDGECIPQHWVCDDEEDCEDGSDERQHCPG
RTCSSSQFTCTNGACIPGGYRCDRVPDCLDGADERNCRKYAVVYPECSELRCANGACY
NRSQRCDQVLNCRDGSDEANCTRRCSSGQFQCSNGECIPRSYVCDHDDDCGDRSDEQN
CTYPTCRGTYFTCPSGRCIHQVWLCDGEDDCEDNADERGCDNVQRECYPGEWPCPSSG
VCIPLEHLCDGTSHCPDGEDETNITAGRNCSIWRCASLSCEHHCHSSPAGGTCSCPLGYM
VSRNDSRSCIDYDDCSLWGMCDQLCEDRTGSHRCSCRDAGVPSLIFSNGRDLLIADVHG
HNARTLVQSQNRGVAVGVDFHFQLQRVFWTDTIQNKVFSVDMDGSHLQVVLNVSVDY
PENLAVDWVNNKLYVVEASVNRIDMVDLDGSNRVTLIAEHLGNPRGLALDPTVGYLFF
SDWDTLNGEPGLERAFMDGTNRYGIIRTKLGWPAGITLDLEAKRVYWVDSRYDYIETTT
YDGLHRKTVVHGGSVIPHPFGITLFEHSVYYTDWTKMAVMKANKYSDNSPQELYRTSQ
RPHGLTVVHAYRQPFVSNPCGTNRGGCEHICVLSHRTDNGGLGYRCRCRMGFDLHADG
KRCMSVRQFLLFSSQLAVRGIPFNLSTQEDIILPITGTPSYFVGVDFSAADNSIFFSDTVKD
IIYKQKIDGTGREVLAANRVDGAEDLAYDWISKNLYWTDPRYRSISVMKVADKTRRAIV
RNLNNPRSIVVHPVAGAMRLFWVDAFFDKIEHSNFDGQNRLSLDRITQISHPFGLTVFGG
YVYFTDWRLGGIVRVRKTDGGEMMIIRRGISHIMHVKSFNADSQIGSNFCNRQTNPNGD
CSHFCFPAPYSQRVCGCPYGMKLEANQQTCVDDPSNEPPTLQCGSNSFSCTNGKCVPQS
YQCDSVDDCHDNSDEANCGANNNTCSPIAFTCANQRCVPRSWHCDGHNDCFDGSDER
DCPTQTPGTCQADQFSCANHHCIPRTWLCDTDNDCGDGSDENNCDSMGTCHPDQFQCP
DHRCIDPNYVCDGDRDCADGADEQGCVYNCTAYEFKCANGHQCVNSYYRCDGVFDC
NDRSDESGCPTRPPGMCHHQSEFQCQSDGSCVPSNWECDGHPDCEDGSDEHHACPPRT
CPTAQFRCDNGNCVFRGWICDGDNDCRDGSDERDCPTPPFRCPSWQWQCPGHSVCVNL
SRVCDNTPDCPNGADESPLCNQESCSDNNAGCTHGCIQGPFGAQCTCPIGFQLSNDSKTC
EDIDECRPPGICSQHCFNERGSFRCHCQEGYTLEADGRTCKASVLRQILVEWSHNGTPEA
NWTFTAGSKAASRITTDKRNNIYIVSDDILAQPNIIRSLVRDGRNIVAVDFDSVTDLTVTE
SLAVDWVGRNLYWTDYILETIEVSKLDGSHRVVLISENVTNPRGLVVDPRNNSHLMFW
TDWGRNPRIERASMDGKLRTTIISSKLYWPNGLTIDYPNNLLYFADAYLDFIDYCDYDG
KNRKQVLASDLVLQHPHAITIFEDFVYWTDRYVNRVIRANKWHGQNQTVMLYNLPQP
MGLVALHPARQPAGYNPCDPRSSPCTHICLLSAVGPRFYSCACPSGWTLAADQFTCARV
EDPFLVVVRDSIIYGIPLNPNDKSNDAMVPVAGLLNGYDVDFDDAEQMIYWVEHPGEIH
RVKSDGTNRTEFAPAAILGSPVGLALDWMSQNLYYSNPASQSIEVLKLKGEVQYRKTLI
TNNGSPTGAGAPAGIAVDPARGKMYWTDQGTESGIPAKVASADMDGSNAAILFTHNLE
HVEFITIDIRENKLYWAVTGTGVIERGDPDGSNRITMVNGLSHPWGVAVYDSFLYFTDR
DFEVIERVDKATGLNRVVMRDNVAASAGSSNGCSNNMGTCQQLCLPRPGGLFSCACAT
GFKLSADNRTCSPYQSYVVISMLTAIKGFSLEGADHSESMVPVAGRGRNALHVDVHMS
SGFIYWCDFSSTVASQNGIRRIKPDGSGFRSIVTSGIGRNGIRGIAVDWAAGNLYFTNAFL
TETYIEVIRLNTTFRRVLLKTQVDMPRHIVVDPMNRYLFWADYGQTPKIERAFLDGSNR
TVLVSSGIVTPRGLALDHRDGYIYWVDDSLDMIARVRPDGGETEVVRYGSRYPTPYGVT
VFEGNVIWVDRNLKKVFQASKQPGATDQPLVIRDNINMLRDVTIFDRRMQPSSAHELNN
NPCLESNGGCAHFCFAIPGSQTRKCSCAFGNLAADSSSCVVSRDDYLIYTTESTVRSLRL
DPEDHSLPFPVVNVPRTSVALDFDRLDGRIYFTQSSGVGQSKISFITLASPTSPATEVASAP
DGIAYDWINKRIYYSDYINQSISSMAVDGSQRTVVAQVPRPRAIMLDPCRGYMYWTDW
GTSAKIERATLGGNFRTEIVNTSLVWPNGLTLDYDEQRLYWADASLQKIERCSLTGTNR

EVIVSTAIYPFAMTVYGQHIYWTDWNTRSIYRANKHDGSDQRVMLQNLPSRPMDIHVL
SNSKQQQCSSPCEQFNGGCSHICAPGPQGAECQCPSEGRWYLADNKHCIPDNGTRCQSG
QFTCMNGRCIRAQWKCDNDDDCGDGSDELERVCAFHTCEPTVFTCGNGRCVPYHYRC
DHYNDCGDNSDETGCIFRPCDPNTEFTCNNGRCIAREYVCNGMNNCYDNGTSDEQNCA
ERTCQPEHTKCQTTNICIPRSYLCDGDNDCGDNSDESPTHCATSTCSQNEFRCSSGRCIPG
HWYCDGGTDCSDGSDEPVTCNMALGKVALLTAVCFIPAATVVRTCNSDQFRCDDGRCI
ASSWICDGDNDCGDMSDEDERHSCANRTCFPQEFTCINNRPPQRKCIPRDWVCDGDAD
CSDAYDEHQNCTRRSCTANEFTCNNGLCIRNSYRCDRRNDCGDSSDEQGCTYQPCQQH
QFTCQNGRCVSQDFVCDGDNDCGDESDELDHLCRTPAPTCPPGNFRCDNGNCIPLSEVC
DRNDDCNDNSDEKGCDPSMHHCDHNCTDTPTSFICTCRPGFRLMSDNKTCDDVDECSV
TPSVCSQVCENTMGSYVCKCAPGFLREPDGRSCRQNSNISPYLIFSNRYYLRNLSTDGEA
YSLILQGLTSVVALDFDRVDRRLYWIDVSRRVLERMFFNGTGREVVVNGILHGEGLAVD
WVGRKLYWVDSFLDCMKVSELDGRFVRKLAEHCVDANNTYCFENPRAIVLHPKFGYV
YWTDWGDKAFIGRVGMDGNNKSAIITTKIEFSDLDGNHRHTVYDGVLPHPFAITVFEES
VYWTDWNTRTVEKGNKYNGSGREALVNTTHRPFDIHVCHPYRQPIVTNPCAVNNGGC
SHLCLLRAGGQGFTCECPDHFLTVQIGGAARCLPMCSSTQYRCADNERCIPIWWKCDGQ
RDCRDGSDEPYTCPVRHCRLGQFQCNDGNCTSPHFLCNSNQDCPDGSDEDAVLCATHQ
CESHQWQCANKRCISEAWQCDGENDCGDGSDEDPAHCSSRTCRPGQFKCRNGRCIPQS
WKCDVDDDCGDNSDEPIEECMGPAYRCDNHTEFDCRTNYRCVPLWAVCNGHNDCRD
NSDEQNCEELTCEPAGDFRCDNHQCIPLRWRCDGDNDCGDGSDERNCTPRPCTESEYRC
DNLHCIPDRWVCDHDNDCEDNSDERDCELRTCHPGYFQCGSGHCISERFKCDGNADCL
DFTDESSCPTRYPNSTYCPPFLFECKNHVCVQQHWICDGDNDCGDNSDEELHLCLDISC
DPPFRFRCDNTRCIYSHELCNSIDDCGVTPTHGPCTEDEYKCGNGQCIPLQYACDDYDD
CEDQSDELGCYYGHGRTCSENLCEHNCTDLSAGGFICSCRPGYKPNPEDKNSCNDVNEC
EVYGTCPQLCRNTKGSYECFCADGFRSVGEQPGVECAAEGNPPVLLLPDNVRIRRFNLS
SAQYSDYVDNAEHIQALDYLWDPEGLGLSIVYWTVLGRGSEFGAIKRAYMTTFDDHGN
NPVKEVDLNLRYISSPDGIAVDWIGGHIYWTDAGTNRIEVSKLDGRYRKWLIHSDLDQP
AAIVMFWTDWGRKPKIETAWMDGQHREVLLDEDLGWPTGLALDYLNENRIYWCDSKE
NIIESMKADGTDRQMIISGDIGHPYSLDVFEGHVYWTTKEKGEVWKKDKFGKGDKVKV
LTINPWLTQVRIYQQHRHNHSVLNPCQGVCSHLCLLRPGGYTCACPQGSTLLTFNKNEC
DAAIEAEVSMPLACRCMNGGTCYTDEGGLPKCPYGYSGSFCEMGRSRAAPAGTAVTVL
LAVVIILITGALVVGVFLNYKRTGSLIPSMPKLPSLSSLVKSADTGNGVSFHSGDNVTMD
LEPQTLGVSFIDRAMQLDENFADSGRQPITFENPLYSTAAGPSSDPAVIHATQVTVNVSG
DQVENNFSNPVFNAHEQAVEVKSRPAELTTTEESKWSFFKRKLKPSTTFENPTYSEMQD
EQTRGAADASSSSQPSPFVPPPKPQKREKLSAYSPTEDTFTDTANLVKEDIIFVYLCTDAA
EGECYEYFSF

> Truncated LRP2 protein by the premature stop codon (2,204 AA)
MERPTVRITQTKLAAVTQPTCESAQFQCLSDGECIPQHWVCDDEEDCEDGSDERQHCPG
RTCSSSQFTCTNGACIPGGYRCDRVPDCLDGADERNCRKYAVVYPECSELRCANGACY
NRSQRCDQVLNCRDGSDEANCTRRCSSGQFQCSNGECIPRSYVCDHDDDCGDRSDEQN
CTYPTCRGTYFTCPSGRCIHQVWLCDGEDDCEDNADERGCDNVQRECYPGEWPCPSSG
VCIPLEHLCDGTSHCPDGEDETNITAGRNCSIWRCASLSCEHHCHSSPAGGTCSCPLGYM
VSRNDSRSCIDYDDCSLWGMCDQLCEDRTGSHRCSCRDAGVPSLIFSNGRDLLIADVHG
HNARTLVQSQNRGVAVGVDFHFQLQRVFWTDTIQNKVFSVDMDGSHLQVVLNVSVDY
PENLAVDWVNNKLYVVEASVNRIDMVDLDGSNRVTLIAEHLGNPRGLALDPTVGYLFF

SDWDTLNGEPGLERAFMDGTNRYGIIRTKLGWPAGITLDLEAKRVYWVDSRYDYIETTT
YDGLHRKTVVHGGSVIPHPFGITLFEHSVYYTDWTKMAVMKANKYSDNSPQELYRTSQ
RPHGLTVVHAYRQPFVSNPCGTNRGGCEHICVLSHRTDNGGLGYRCRCRMGFDLHADG
KRCMSVRQFLLFSSQLAVRGIPFNLSTQEDIILPITGTPSYFVGVDFSAADNSIFFSDTVKD
IIYKQKIDGTGREVLAANRVDGAEDLAYDWISKNLYWTDPRYRSISVMKVADKTRRAIV
RNLNNPRSIVVHPVAGAMRLFWVDAFFDKIEHSNFDGQNRLSLDRITQISHPFGLTVFGG
YVYFTDWRLGGIVRVRKTDGGEMMIIRRGISHIMHVKSFNADSQIGSNFCNRQTNPNGD
CSHFCFPAPYSQRVCGCPYGMKLEANQQTCVDDPSNEPPTLQCGSNSFSCTNGKCVPQS
YQCDSVDDCHDNSDEANCGANNNTCSPIAFTCANQRCVPRSWHCDGHNDCFDGSDER
DCPTQTPGTCQADQFSCANHHCIPRTWLCDTDNDCGDGSDENNCDSMGTCHPDQFQCP
DHRCIDPNYVCDGDRDCADGADEQGCVYNCTAYEFKCANGHQCVNSYYRCDGVFDC
NDRSDESGCPTRPPGMCHHQSEFQCQSDGSCVPSNWECDGHPDCEDGSDEHHACPPRT
CPTAQFRCDNGNCVFRGWICDGDNDCRDGSDERDCPTPPFRCPSWQWQCPGHSVCVNL
SRVCDNTPDCPNGADESPLCNQESCSDNNAGCTHGCIQGPFGAQCTCPIGFQLSNDSKTC
EDIDECRPPGICSQHCFNERGSFRCHCQEGYTLEADGRTCKASVLRQILVEWSHNGTPEA
NWTFTAGSKAASRITTDKRNNIYIVSDDILAQPNIIRSLVRDGRNIVAVDFDSVTDLTVTE
SLAVDWVGRNLYWTDYILETIEVSKLDGSHRVVLISENVTNPRGLVVDPRNNSHLMFW
TDWGRNPRIERASMDGKLRTTIISSKLYWPNGLTIDYPNNLLYFADAYLDFIDYCDYDG
KNRKQVLASDLVLQHPHAITIFEDFVYWTDRYVNRVIRANKWHGQNQTVMLYNLPQP
MGLVALHPARQPAGYNPCDPRSSPCTHICLLSAVGPRFYSCACPSGWTLAADQFTCARV
EDPFLVVVRDSIIYGIPLNPNDKSNDAMVPVAGLLNGYDVDFDDAEQMIYWVEHPGEIH
RVKSDGTNRTEFAPAAILGSPVGLALDWMSQNLYYSNPASQSIEVLKLKGEVQYRKTLI
TNNGSPTGAGAPAGIAVDPARGKMYWTDQGTESGIPAKVASADMDGSNAAILFTHNLE
HVEFITIDIRENKLYWAVTGTGVIERGDPDGSNRITMVNGLSHPWGVAVYDSFLYFTDR
DFEVIERVDKATGLNRVVMRDNVAASAGSSNGCSNNMGTCQQLCLPRPGGLFSCACAT
GFKLSADNRTCSPYQSYVVISMLTAIKGFSLEGADHSESMVPVAGRGRNALHVDVHMS
SGFIYWCDFSSTVASQNGIRRIKPDGSGFRSIVTSGIGRNGIRGIAVDWAAGNLYFTNAFL
TETYIEVIRLNTTFRRVLLKTQVDMPRHIVVDPMNRYLFWADYGQTPKIERAFLDGSNR
TVLVSSGIVTPRGLALDHRDGYIYWVDDSLDMIARVRPDGGETEVVRYGSRYPTPYGVT
VFEGNVIWVDRNLKKVFQASKQPGATDQPLVI

Supplementary Text S2: shell scripts involved in this study

```
#QC of raw genomic sequence data#
dir="/data/ljy/Raw_data"
id="GoldfishXXX"
fastqc -t 10 ${dir}/fastq/${id}_1.fastq.gz ${dir}/fastq/${id}_2.fastq.gz -o ${dir}/qc_reports
fastp -w 10 -i ${dir}/fastq/${id}_1.fastq.gz -I ${dir}/fastq/${id}_2.fastq.gz -o ${dir}/clean/${id}_1.clean.fastq.gz -
O ${dir}/clean/${id}_2.clean.fastq.gz -h ${dir}/clean/${id}_fastp_report.html
fastqc -t 10 ${dir}/clean/${id}_1.clean.fastq.gz ${dir}/clean/${id}_2.clean.fastq.gz -o ${dir}/qc_clean_reports

#Alignment of genomic sequence data#
dir="/data/ljy/Raw_data"
outdir="/data/ljy/bam"
id="GoldfishXXX"
bwa mem -aM -t 5 -R "@RG\tID:${id}\tPL:ILLUMINA\tLB:${id}\tDS:${id}\tPU:${id}\tSM:${id}"
/data/ljy/Goldfish/Goldfish_genome/Goldfish.v20190830.chr.fasta ${dir}/clean/${id}_1.clean.fastq.gz
${dir}/clean/${id}_2.clean.fastq.gz | samtools view -b -S -h -o ${outdir}/${id}.bam -
samtools sort -T ${outdir}/${id}.sorting ${outdir}/${id}.bam > ${outdir}/${id}.sort.bam; samtools index
${outdir}/${id}.sort.bam

#Calling SNPs and small Indels#
id="GoldfishXXX"
dir="/data/ljy/bam/"
outdir="/data/ljy/GATK/"
genome="/data/ljy/Goldfish/Goldfish_genome/Goldfish.v20190830.chr.fasta"
gatk --java-options "-Xmx50g" HaplotypeCaller -R ${genome} -I ${dir}${id}.sort.bam -O ${outdir}${id}.g.vcf.gz
-ERC GVCF

#Merge GVCF files for SNPs and small Indels#
sd="Goldfish"
gatk --java-options "-Xms50g" CombineGVCFs \
-R /data/ljy/Goldfish/Goldfish_genome/Goldfish.v20190830.chr.fasta \
-V /data/ljy/GATK/GoldfishX.g.vcf.gz \
-V /data/ljy/GATK/GoldfishXX.g.vcf.gz \
-V /data/ljy/GATK/GoldfishXXX.g.vcf.gz \
-L Chr9:19730000-28630000 \
-O /data/ljy/Analysis/${sd}_temp.vcf.gz
gatk --java-options "-Xms50g" GenotypeGVCFs --allow-old-rms-mapping-quality-annotation-data \
-R /data/ljy/Goldfish/Goldfish_genome/Goldfish.v20190830.chr.fasta \
-V /data/ljy/Analysis/${sd}_temp.vcf.gz \
-O /data/ljy/Analysis/${sd}.g.vcf

#Calling SVs#
id="GoldfishXXX"
name="ljy"
samtools view -b -F 1294 /data/${name}/bam/${id}.sort.bam > /data/${name}/lumpy/tmp/${id}.discordants.bam
samtools view -h /data/${name}/bam/${id}.sort.bam \
   | /usr/local/lumpy-sv/scripts/extractSplitReads_BwaMem -i stdin \
   | samtools view -Sb - \
   > /data/${name}/lumpy/tmp/${id}.splitters.bam
samtools sort /data/${name}/lumpy/tmp/${id}.discordants.bam -o
/data/${name}/lumpy/tmp/${id}.sort.discordants.bam
samtools sort /data/${name}/lumpy/tmp/${id}.splitters.bam -o /data/${name}/lumpy/tmp/${id}.sort.splitters.bam
lumpyexpress -B /data/${name}/bam/${id}.sort.bam -S /data/${name}/lumpy/tmp/${id}.sort.splitters.bam -D
/data/${name}/lumpy/tmp/${id}.sort.discordants.bam -o /data/${name}/lumpy/lumpy_${id}.vcf
```

```
#Merge VCF files for SVs#
bcftools merge --force-samples -r Chr9:19730000-28630000 -o /data/ljy/Analysis/Goldfish.vcf \
/data/ljy/lumpy/lumpy_GoldfishX.vcf /data/ljy/lumpy/lumpy_GoldfishXX.vcf
/data/ljy/lumpy/lumpy_GoldfishXXX.vcf
```

```
#Pooled genomic sequencing sample analysis#
id1="SSC"
id2="SSN"
dir="/data/ljy/bam/"
wsize="50" #kb
ssize="10" #kb
psize="64:80"
psize1="64"
psize2="80"
mkdir ${dir}Fst
samtools mpileup -B ${dir}${id1}.realigned.bam ${dir}${id2}.realigned.bam > ${dir}Fst/${id1}_${id2}.mpileup
java -ea -Xmx7g -jar /usr/share/popoolation2_1201/mpileup2sync.jar --input ${dir}Fst/${id1}_${id2}.mpileup --
output ${dir}Fst/${id1}_${id2}.sync --fastq-type sanger --min-qual 20 --threads 8
perl /usr/share/popoolation2_1201/snp-frequency-diff.pl --input ${dir}Fst/${id1}_${id2}.sync --output-prefix
${dir}Fst/${id1}_${id2} --min-count 4 --min-coverage 12 --max-coverage 400
echo -e "Chromosome\tStart\tEnd\tFeature\tDeltaAF" > ${dir}Fst/${id1}_${id2}_dAF.igv
awk '{if (NR!=1) {print $1,"\t",$2,"\t",$2+1,"\t","snp","\t",$9}}' ${dir}Fst/${id1}_${id2}_pwc >>
${dir}Fst/${id1}_${id2}_dAF.igv
sed -i "s/ //g" ${dir}Fst/${id1}_${id2}_dAF.igv
perl /usr/share/popoolation2_1201/fst-sliding.pl --input ${dir}Fst/${id1}_${id2}.sync --output
${dir}Fst/${id1}_${id2}_w${wsize}k_${ssize}k.fst --min-count 4 --min-coverage 12 --max-coverage 400 --min-
covered-fraction 0.01 --window-size ${wsize}000 --step-size ${ssize}000 --pool-size ${psize}
perl /usr/share/popoolation2_1201/export/pwc2igv.pl --input ${dir}Fst/${id1}_${id2}_w${wsize}k_${ssize}k.fst --
output ${dir}Fst/${id1}_${id2}_w${wsize}k_${ssize}k.igv
```

```
#QC of raw output of GATK HaplotypeCaller#
id="GoldfishXXX"
cd /data/ljy/Analysis
gatk SelectVariants \
    -V ${id}.g.vcf \
    -select-type SNP \
    -O ${id}_snps.vcf
gatk VariantFiltration \
   -R /data/ljy/Genome/GCF_000002315.5_GRCg6a_genomic.fna \
   -V ${id}_snps.vcf \
   -O ${id}_snps_filtered.vcf \
   --filter-expression "QD < 2.0 || FS > 60.0 || MQ < 40.0 || MQRankSum < -12.5 || ReadPosRankSum < -8.0" \
   --filter-name "my_filter"
bgzip -c ${id}_snps_filtered.vcf > ${id}_snps_filtered.vcf.gz
tabix -p vcf ${id}_snps_filtered.vcf.gz
bcftools view -f PASS ${id}_snps_filtered.vcf.gz > ${id}_snp_filtered.g.vcf
```

```
#Phasing the SNPs#
id="Goldfish_1"
java -jar -Xms20g /data/ljy/beagle/beagle.18May20.d20.jar gt=/data/ljy/Analysis/${id}_snp_filtered.g.vcf
out=/data/ljy/Analysis/${id}_phased.g.vcf
```

```
#Calculate the pair-wise genetic distances#
id="Goldfish_1"
```

python3 /data/ljy/genomics_general-master/distMat.py -g /data/ljy/Analysis/${id}_phased.g.vcf -f phased --windType cat -o /data/ljy/Analysis/${id}.dist

#Analysis of transcriptomic sequence data#
dir="/data/ljy/RNA/"
genome="/data/ljy/Goldfish/Goldfish_genome/Goldfish.v20190830.chr.fasta"
ht2="/data/ljy/Genome_Goldfish/Goldfish"
annot="Goldfish.v20190830.chr.gff3"
ID="GoldfishX GoldfishXX GoldfishXXX"
cd ${dir}
mkdir -p clean sam_bam bam qc_reports gtf
for id in $ID; do
fastqc -t 10 ${dir}/fastq/${id}_1.fastq.gz ${dir}/fastq/${id}_2.fastq.gz -o ${dir}/qc_reports
fastp -w 10 -l 30 -n 15 -i ${dir}/fastq/${id}_1.fastq.gz -I ${dir}/fastq/${id}_2.fastq.gz -o ${dir}/clean/${id}_1.clean.fastq.gz -O ${dir}/clean/${id}_2.clean.fastq.gz -h ${dir}/clean/${id}_fastp_report.html -j ${dir}/clean/${id}_fastp_report.json
fastqc -t 10 ${dir}/clean/${id}_1.clean.fastq.gz ${dir}/clean/${id}_2.clean.fastq.gz -o ${dir}/qc_reports
hisat2 --dta -p 10 -x ${ht2} -1 ${dir}/clean/${id}_1.clean.fastq.gz -2 ${dir}/clean/${id}_2.clean.fastq.gz -S ${dir}/sam_bam/${id}.sam
samtools view -b -S -h ${dir}/sam_bam/${id}.sam > ${dir}/sam_bam/${id}.bam
samtools sort -T ${dir}/sam_bam/${id}.sorting ${dir}/sam_bam/${id}.bam > ${dir}/bam/${id}.bam
samtools index ${dir}/bam/${id}.bam
stringtie -f 0.05 -m 150 -A ${dir}/gtf/${id}_gene_abun.tab -b ${dir}/gtf/${id}/ -e -p 5 -G ${annot} ${dir}/bam/${id}.bam -o ${dir}/gtf/${id}_stringtie.gtf
done;
cd /data/ljy/RNA/
/data/ljy/stringtie-2.2.1.Linux_x86_64/prepDE.py3 -i /data/ljy/RNA/sample_list.txt -l 150
